# Targeting DDOST improves the efficacy of lenvatinib and immunotherapy in hepatocellular carcinoma

**DOI:** 10.1038/s12276-025-01597-9

**Published:** 2025-12-19

**Authors:** Jun Pu, Jingjing Ma, Yan Liu, Rongrong Cui, Yao Yao, Guanjun Zhang, Peng Hou, Xi Liu, Qi Yang, Meiju Ji

**Affiliations:** 1 Department of Pathology, The First Affiliated Hospital of Xi’an Jiaotong University, Xi’an, China; 2 Department of Endocrinology and International Joint Research Center for Tumor Precision Medicine of Shaanxi Province, The First Affiliated Hospital of Xi’an Jiaotong University, Xi’an, China; 3 Center for Translational Medicine, The First Affiliated Hospital of Xi’an Jiaotong University, Xi’an, China

**Keywords:** Cancer, Cell biology

## Abstract

Hepatocellular carcinoma (HCC) remains one of the most lethal malignancies, with limited efficacy of systemic therapies due to poor survival benefit and drug resistance. Dolichyl-diphosphooligosaccharide-protein glycosyltransferase noncatalytic subunit (DDOST), a critical component of oligosaccharyltransferase (OST), is upregulated in multiple cancers, yet its role in HCC is unclear. Here we demonstrate that DDOST expression is elevated in HCC tissues and correlated with poor prognosis. Functional studies showed that *DDOST* knockdown suppressed cell proliferation, induced cell cycle arrest and enhanced their lenvatinib sensitivity both in vitro and in vivo. Mechanistically, DDOST depletion impaired EGFR N-glycosylation, suppressing downstream AKT, ERK5 and ERK1/2 signaling, thereby sensitizing HCC cells to lenvatinib. Loss of DDOST also reduced PD-L1 glycosylation. Furthermore, the OST inhibitor NGI-1 and NGI-1-loaded nanoparticles exerted potent antitumor effects and further augmented the efficacy of lenvatinib and immunotherapy. These findings highlight DDOST as a promising therapeutic target to improve treatment outcomes in HCC.

## DDOST targeting enhances lenvatinib efficacy in HCC

Primary liver cancer, particularly hepatocellular carcinoma (HCC), is a major global health issue. Despite advances in treatment, many patients develop resistance to drugs such as lenvatinib, a common therapy. Researchers explored how a protein called DDOST might contribute to this resistance. They studied HCC cells and found that DDOST is often overactive in these cancers, helping the cancer cells survive and resist treatment. By reducing DDOST activity, they observed that cancer cells became more sensitive to lenvatinib. This was because DDOST affects a process called N-glycosylation, which is crucial for the activity of proteins such as EGFR and PD-L1 involved in cancer growth and immune evasion. The study suggests that targeting DDOST could improve the effectiveness of existing treatments for HCC. In the future, therapies that inhibit DDOST might enhance the response to both lenvatinib and immunotherapy.

This summary was initially drafted using artificial intelligence, then revised and fact-checked by the author.

## Introduction

Primary liver cancer, particularly hepatocellular carcinoma (HCC), is among the most prevalent malignancies worldwide^1^. As the sixth most common and the third deadliest cancer worldwide, HCC poses a significant threat to public health^2^. Tyrosine kinase inhibitors (TKIs) and immune checkpoint inhibitors, which are critical systemic therapies in HCC, have profoundly transformed HCC management over the past decades^3^. At present, about 50–60% of patients with HCC in advanced stages receive systemic therapies^4^. While the use of atezolizumab (an anti-PD-L1 antibody) and bevacizumab (an anti-VEGF antibody) has improved the treatment of HCC^5^, multikinase inhibitors (mTKIs) continue to play a vital role in certain cases, providing therapeutic diversity and flexibility.

Lenvatinib was approved by the US Food and Drug Administration for first-line treatment of unresectable HCC in 2018^6^. It is an oral mTKI targeting FGFR1-4, PDGFRα, RET, VEGFR1-3 and KIT^7^, offering a stronger objective response rate than sorafenib^8^. Lenvatinib has shown promising results in clinical use; however, its effectiveness is hindered by resistance mechanisms that are not yet fully understood. EGFR overactivation occurs in over 60% of patients with HCC^9^. A previous study demonstrated that the activity of EGFR was significantly increased when cells acquired resistance to lenvatinib^10^. Also, a recent study using CRISPR–Cas9 screens showed that feedback activation of the EGFR–PAK2–ERK5 signaling pathway reduced the sensitivity of lenvatinib^11^. In addition, the activation of the EGFR-mediated STAT3–ABCB1 pathway has been involved in enhanced lenvatinib exocytosis in lenvatinib-resistant HCC cells^12^. These observations underscore the therapeutic potential of targeting EGFR to overcome lenvatinib resistance and improve clinical outcomes.

N-linked glycosylation is necessary for the proper functioning of membrane associated proteins^13^, including EGFR, as it facilitates the protein sorting, intracellular transport and ligand binding of EGFR^14,15^. Experimental inhibition of N-linked glycosylation can decrease receptor surface expression and impair its downstream signaling pathways by disrupting the above bioprocesses, thereby potentiating the antitumor efficacy of lenvatinib^16^. PD-L1, like EGFR, is highly dependent on N-linked glycosylation for both its stability and function. Its glycosylation is crucial for its interaction with PD-1^17^, a process that most often occurs for tumor cells to evade immune defenses. Moreover, the presence of N-glycans prevents the recognition of PD-L1 by both diagnostic and therapeutic antibody such as atezolizumab^18^. This leads to a failure to accurately estimate the response of PD-(L)1 inhibitors in patients with HCC^19,20^. Blocking the N-glycosylation pathway of PD-L1 has been proven to enhance the efficacy of immunotherapy in several solid tumors by reducing PD-L1 stability and augmenting immune recognition^21–25^. Based on this information, targeting N-linked glycosylation enhances the efficacy of both mTKIs and PD-(L)1 inhibitors in HCC.

The transfer of lipid-linked oligosaccharide (LLO) to asparagine side chains in nascent proteins, catalyzed by OST, is a key step in the N-glycosylation pathway^26^. OST is a membrane-embedded protein complex in the endoplasmic reticulum (ER) that comprises the catalytic subunits STT3A/STT3B as well as several noncatalytic subunits^27^. This process is requisite for the N-glycosylation of most membrane and secretory proteins, including PD-L1 and EGFR^25,28–30^.

DDOST as a key subunit of OST is upregulated in various human cancers^31–34^. High expression of DDOST has been linked to poor patient survival and increased malignancy in cancers such as cutaneous squamous cell carcinoma and glioblastoma^31,32^. Several studies have highlighted the potential for targeting DDOST in cancer therapy. Inhibiting DDOST could disrupt the glycosylation of critical proteins, thereby impairing tumor growth and enhancing immune recognition^35,36^. However, whether DDOST participates in the N-glycosylation pathway of EGFR and PD-L1, as well as its definitive biological functions and underlying molecular mechanisms in HCC, remains unclear. This study aims to confirm the effects of DDOST on malignant behaviors, drug resistance and antitumor immunity and illustrate related molecular mechanisms in HCC, thereby providing an effective therapeutic potential strategy for HCC and improving survival rates of patients with HCC.

## Materials and methods

### Clinical samples

With approval of the institutional review board and patient consent, we collected 24 pairs of HCC tissues and adjacent noncancerous liver tissues (control subjects) from the First Affiliated Hospital of Xi’an Jiaotong University. All samples were stored at −80 °C. In addition, eight pairs of paraffin-embedded HCC tissues and their control subjects were obtained from Department of Pathology. The histological diagnosis of all HCC tissues was confirmed by a senior pathologist at the hospital.

### Cell culture and drug treatments

Human HCC cell lines (MHCC97H, Huh7, Li-7 and SNU387) were obtained from Meisen CTCC (Zhejiang, China) and National Collection Authenticated Cell Cultures (Shanghai, China). The murine HCC cell line H22 was purchased from the China Center for Type Culture Collection (Wuhan, China). Cells were cultured at 37 °C with 5% CO_2_ in proper medium (RPMI-1640 or DMEM) supplemented with 10% fetal bovine serum. Cells were exposed to OST inhibitor NGI-1 (Selleck, cat. no. S8750), lenvatinib (TOPSCIENCE, cat. no. T8541) or tunicamycin (TM, Selleck, cat. no. S7894) at the specified concentrations and durations in some experiments.

### Quantitative reverse transcription PCR (qRT–PCR)

The extraction of total RNA, cDNA preparation and qRT–PCR assays were performed as previously described^37^. The primer sequences are presented in Supplementary Table 1. The mRNA expression of targeted genes was normalized to *β-Actin*.

### Protein extraction and western blotting analysis

Cells were cultured and treated as indicated, then lysed in prechilled RIPA buffer with protease inhibitors. HCC tissues were homogenized in the same buffer. Equal protein amounts were subjected to western blotting analysis according to previously described protocols^38^. Quantification of key protein expression was done using ImageJ. The primary antibodies used are presented in Supplementary Table 2.

### Immunohistochemistry (IHC)

IHC staining was conducted to assess protein levels following established protocols^39,40^. The primary antibodies are also presented in Supplementary Table 2. Quantification of stained sections was performed using ImageJ as previously described^41^.

### Immunofluorescence (IF)

For IF staining, paraffin-embedded tissue sections were deparaffinized, rehydrated and subjected to antigen retrieval. Cultured cells grown on coverslips were washed with phosphate-buffered saline (PBS) containing 0.3% Triton X-100, followed by fixation with 4% paraformaldehyde. Samples were blocked in goat serum for 30 min and incubated with the corresponding primary antibodies. After PBS washes, sections or coverslips were treated with fluorescence-conjugated secondary antibodies. Nuclear staining was performed with DAPI (C1002, Beyotime) before mounting. Images were acquired using a Leica fluorescence microscope, and fluorescence intensity was quantified with ImageJ software. Details of antibodies used in IF assays are provided in Supplementary Table 2.

### Knockdown and ectopic expression of *DDOST*

Small interfering (si)RNA) oligonucleotides (si-DDOST-1, si-DDOST-2 and si-DDOST-3) and control siRNA (si-NC) were purchased from RiboBio, and the siRNA sequences are presented in Supplementary Table 3. Lentivirus encoding short hairpin (sh)RNA targeting *DDOST* (sh-DDOST-1, sh-DDOST-2 and sh-DDOST-3) or control shRNA (sh-NC) were purchased from GeneChem. The shRNA sequences are presented in Supplementary Table 4. To knock down *DDOST*, cells were transfected with 60 nM siRNAs in siRNA Transfection Reagent (Roche Diagnostics) at ~40% confluence for 48 h, or infected with lentivirus at a multiplicity of infection of 10–20 as per the manufacturer’s instructions. The plasmid expressing 3×FLAG-tagged DDOST and empty vector were obtained from Miao Ling Biotechnology. HCC cells were transfected with the plasmids using DNA Transfection Reagent (Thermo Fisher Scientific) at ~70% confluence.

### MTT assay

*DDOST*-knockdown or -overexpressing HCC cells, along with their control cells, were plated in 96-well plates at 800–1,000 cells per well. In some experiments, HCC cells (1,000–2,000 cells per well) were treated with optimized concentrations of lenvatinib or NGI-1. Cell viability was then assessed using MTT assays at day 0, day 1, day 3, day 5 and day 7^40^. The half-maximal inhibitory concentration (IC_50_) values with 95% confidence intervals were derived from normalized dose–response curves of lenvatinib using GraphPad Prism (version 8.2).

### Colony formation assay

*DDOST*-knockdown or -overexpressing HCC cells, along with their control cells, were plated in 12-well plates at 800–1,500 cells per well. For drug treatment, cells (1,500–3,000 cells per well) were cultured in medium with varying concentrations of lenvatinib or NGI-1 for 10 days, following washing, fixing and then staining with a crystal violet.

### Cell cycle analysis

HCC cells were transfected with siRNAs for 48 h or treated with 10 μM NGI-1 for 24 h. After that, they were fixed with 66% cold methanol for at least 4 h, then washed with PBS and stained with propidium iodide. Finally, the cell cycle distribution was analyzed by flow cytometry.

### ER-LucT reporter assay

The ER-LucT reporter plasmid, which expresses firefly luciferase containing three potential glycosylation sites and an in-frame ER translation leader sequence for N-glycosylation detection, was purchased from Miao Ling Biotechnology. To verify the efficacy of the ER-LucT reporter, HCC cells were transfected with this plasmid and then si-DDOST or treated with NGI-1 or N-linked glycosylation inhibitor TM. After 24 or 48 h, cells were lysed and incubated with medium containing 100 μg/ml Luciferase substrate for 10 min. Bioluminescence was measured using a Bio-Tek Cytation5 multimode microplate reader with 10-s integration time per well, and signal intensity was quantified as total relative light units per well.

### Detection of membrane PD-L1

Following transfection with siRNAs for 48 h or treatment with 10 μM NGI-1 for 24 h, HCC cells were collected and incubated with APC-labeled anti-human PD-L1 antibody (BioLegend, cat. no. 329708) for 20 min, then washed and resuspended in 200 μl PBS. Then, membrane PD-L1 was detected by flow cytometry.

### mRNA sequencing and data analysis

Total RNA was extracted using the RNeasy Mini Kit (Qiagen), with RNA quality assessed by gel electrophoresis and Qubit quantification (Thermo Fisher Scientific). Strand-specific sequencing libraries were prepared using the TruSeq RNA Library Prep Kit (Illumina), and 150-bp paired-end sequencing was performed on an Illumina NovaSeq 6000 platform (Genergy Biotechnology).

Differential gene expression analysis was performed using the limma package, with significantly differentially expressed genes (DEGs) defined as those meeting both thresholds: *P* < 0.05 and |log_2_(fold change)| >2. Functional enrichment analyses (Gene Ontology (GO), Kyoto Encyclopedia of Genes and Genomes, and Reactome pathways) were conducted using the clusterProfiler package, with statistical significance set at *P* < 0.05.

### Preparation and physicochemical characteristics of NGI-1-NPs

NGI-1-loaded nanoparticles (NGI-1-NPs) were prepared using a nanoprecipitation technique as described previously^42^. In brief, methoxy poly(ethylene glycol)-b-poly(D,L-lactic-co-glycolic) acid copolymer (PEG–PLGA, weight-average molecular weight (Mw) PEG = 3,400 Da, Mw PLGA = 12,000 Da) was purchased from Ruixibio and polyethyeleneimine (number-average molecular weight (Mn) ~600) from Aladdin Bio-Chem Technology. NGI-1 was dissolved in dimethyl sulfoxide (DMSO) (50 mg/ml) and mixed with polyethyeleneimine solution at a 1:6 (w:w) ratio. Next, the mixture was added to the polymer solution (10 mg/ml) at a 1:10 (w:w) ratio, followed by vertexing and water-bath sonication. The resulting mixture was added dropwise to stirring water to form drug-loaded NPs by self-assembly. NGI-1-NPs were collected, washed and concentrated using an Amicon filter (Merck Millipore, molecular weight cutoff 100 kDa) and then resuspended in PBS. Control NPs were similarly prepared without the drug. NPs were stored at 4 °C temporarily or at −20 °C after freeze-drying. The average size, zeta potential and morphology of NGI-1-NPs or NPs were characterized as described previously^43^.

### Animal studies

Nude mice (male, 5–6 weeks old) were purchased from GemPharmatech and maintained in specific pathogen-free conditions. To determine the oncogenic effect of DDOST, each mouse in a specific group (five mice per group) was subcutaneously injected with *DDOST*-knockdown MHCC97H cells or control cells (5 × 10^6^ per mouse) to establish xenograft tumor models. Moreover, to determine the sensitizing effect of *DDOST* knockdown on lenvatinib, mice were divided into four groups (five mice per group). Next, mice in two groups were subcutaneously injected with *DDOST*-knockdown MHCC97H cells (5 × 10^6^ per mouse), while mice in the other two groups were subcutaneously injected with control cells (5 × 10^6^ per mouse). When tumors reached 40–60 mm^3^, lenvatinib (5 mg/kg) or DMSO was administered daily by gavage. To evaluate the combined effect of NGI-1 and lenvatinib, mice were subcutaneously injected with MHCC97H cells (5 × 10^6^ per mouse) and divided into four groups. Next, these mice received NPs or NGI-1-NPs (NGI-1 ~10 mg/kg) every other day and lenvatinib (5 mg/kg) or DMSO daily by gavage.

To assess the effects of NGI-1-NPs on tumor immunity in BALB/c mice, H22 cells (2 × 10^6^ per mouse) were used to establish subcutaneous tumors. H22 tumor-bearing mice were used with PBS, NGI-1-NPs (NGI-1 ~10 mg/kg, every other day), aPD-1 (100 μg per dose, 3 doses), aPD-L1 (100 μg per dose, 3 doses), NGI-1-NPs + aPD-1 (NGI-1 ~10 mg/kg, every other day; aPD-1: 100 μg per dose, 3 doses) and NGI-1-NPs + aPD-L1 (NGI-1 ~10 mg/kg, every other day; aPD-L1: 100 μg per dose, 3 doses), respectively (8 mice per group). Tumor sizes were measured, and tumor volumes were calculated using the formula: length × width^2^ × 0.5). Mice were euthanized at the end of the experiment. Also, xenograft tumors were isolated, weighed and stored for the following experiments.

### Biosafety evaluation of NGI-1-NPs

To evaluate the biosafety of NGI-1-NPs, we measured serum levels of creatinine (CRE), aspartate aminotransferase (AST), alanine transaminase (ALT) and blood urea nitrogen (BUN) in mice from different experimental groups using commercial assay kits (Nanjing Jiancheng Bioengineering Institute) following the manufacturer’s protocols. In addition, hematoxylin and eosin (H&E) staining was performed to assess pathological changes in the liver, kidney, lung, spleen and heart tissues.

To further investigate the hematologic toxicity of NGI-1-NPs, mice treated with NPs or NGI-1-NPs were euthanized, and peripheral blood was collected for complete blood count analysis. Long bones were isolated, and bone marrow smears were prepared using standard techniques, followed by Wright–Giemsa staining for microscopic evaluation. Two independent pathologists blinded to the treatment groups analyzed all stained sections.

### Assessment of immune infiltration

H22 cell-derived xenograft tumors (three tumors per group) were dissociated to prepare single-cell suspensions. After that, 10^6^ cells per sample were incubated with indicated antibodies for 30 min. PE-labeled anti-mouse CD45 antibody (cat. no. 147711), APC-labeled anti-mouse CD3 antibody (cat. no. 100236), FITC-labeled anti-mouse CD4 antibody (cat. no. 100405) and FITC-labeled anti-mouse CD8 antibody (cat. no. 100705) were purchased from BioLegend. Flow cytometry was then performed to analyze T lymphocyte infiltration.

### Enzyme‑linked immunosorbent assay (ELISA)

Tumor tissues were weighted and homogenized in PBS. The Mouse Granzyme B/IFN-γ ELISA Kit (Fankewei) was used to measure the levels of Granzyme B and IFN-γ according to their instructions. The values were normalized to tissue weight.

### Statistical analysis

Statistical comparisons between two groups were performed using Student’s *t*-test in GraphPad Software (GraphPad Prism Software Version 8.2). Two-way analysis of variance (ANOVA) was used for curve analysis, and differences in Kaplan–Meier survival curves were analyzed by log-rank test. Data are expressed as mean ± s.d., and *P* values <0.05 were considered statistically significant.

## Results

### *DDOST* is upregulated in HCC tissues and related to decreased patient survival

**Fig. 1:**
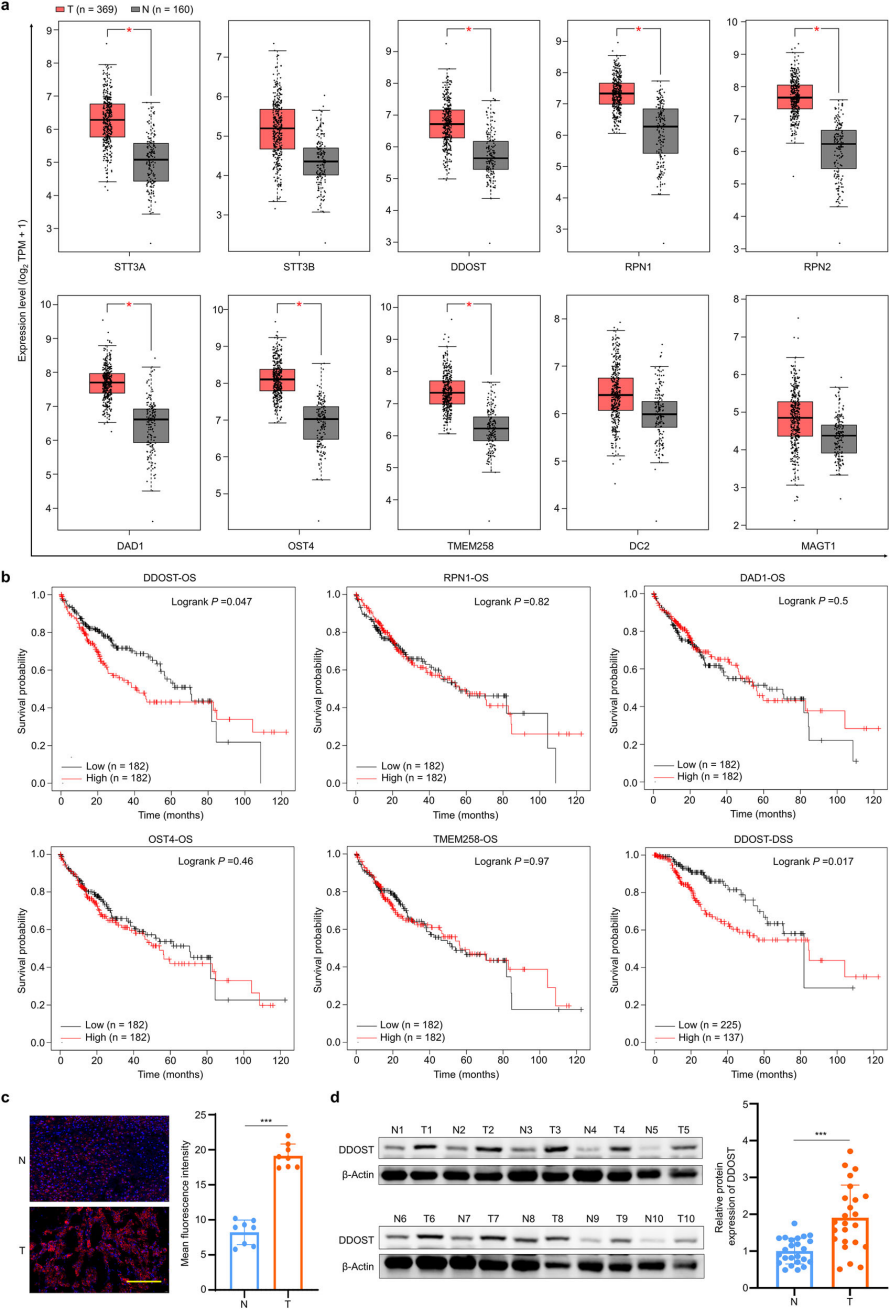
Correlation between increased expression of *DDOST* and poor prognosis in patients with HCC. **a** The mRNA expression of OST subunits in HCC tissues (T) and noncancerous liver tissues (N) (data from TCGA database). **b** Survival analysis of patients with HCC stratified by expression levels of OST subunits using TCGA cohort data. **c** IF assay evaluating DDOST expression in HCC tissues (T, *n* = 8) and matched noncancerous liver tissues (N, *n* = 8). Left: representative images (400×) of IF staining. Right: statistical results. Scale bar, 100 μm. **d** Left: western blotting analysis of DDOST expression in HCC tissues (T, *n* = 24) and matched noncancerous liver tissues (N, *n* = 24). β-Actin served as a loading control. Right: statistical analysis of DDOST proteins levels. Data are expressed as mean ± s.d. *P < 0.05, ***P < 0.001 (unpaired two-tailed Student’s *t*-test for **a**, log-rank test for **b** and paired two-tailed Student’s *t*-test for **c** and **d**).

To investigate the roles of the OST complex members in the tumorigenesis and progression of HCC, RNA-seq data from The Cancer Genome Atlas (TCGA) database was analyzed firstl. We found that the expression of most OST subunits (STT3A, RPN1, DDOST, RPN2, DAD1, OST4 and TMEM258) was significantly upregulated in HCC tissues compared with control subjects (Fig. 1a). Given the previously reported oncogenic roles of STT3A^25,28^ and RPN2^44^, we investigated the correlation of the expression of other noncatalytic subunits (DDOST, RPN1, DAD1, OST4 and TMEM258) with the prognosis of patients with HCC. Our data demonstrated that both overall survival (OS) and disease-specific survival (DSS) were diminished in HCCs with elevated *DDOST* expression (Fig. 1b). Also, we confirmed elevated protein (Fig. 1c,d) and mRNA (Supplementary Fig. 1) expression of *DDOST* in freshly resected HCC tissues compared with their matched control tissues using qRT–PCR, IF and western blotting assays. Next, GO enrichment analysis indicated that DDOST is involved in protein modification and folding, cell cycle progression, DNA replication, and antigen processing and presentation (Supplementary Fig. 2). These data, taken together, suggest that DDOST probably plays an oncogenic role in HCC.

### *DDOST* knockdown inhibits the malignant behaviors of HCC cells and enhances their response to lenvatinib

**Fig. 2:**
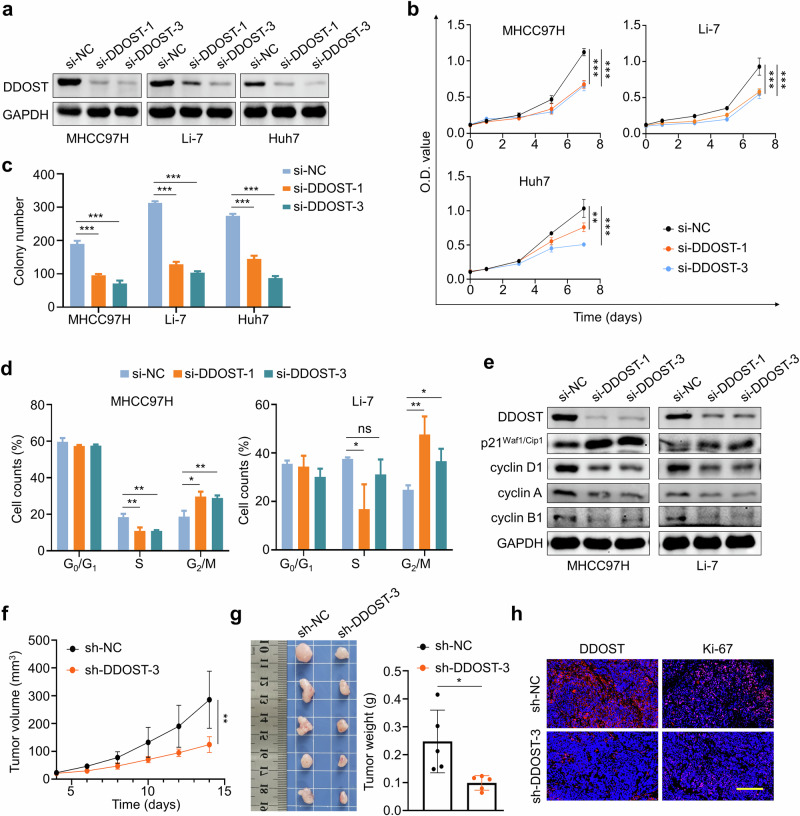
Oncogenic roles of DDOST in HCC cells. **a** Western blotting analysis was performed to validate the knockdown of *DDOST* by two siRNAs in MHCC97H, Li-7 and Huh7 cells. GAPDH was used as a loading control. **b** MTT assays showing the inhibitory effect of *DDOST* knockdown on the proliferation of MHCC97H, Li-7 and Huh7 cells. **c** The inhibitory effect of *DDOST* knockdown on colony formation ability of the above cells. **d** Flow cytometry analysis demonstrating cell cycle arrest induced by *DDOST* knockdown in MHCC97H and Li-7 cells. **e** Western blotting analysis of key proteins associated with cell cycle transition in *DDOST*-knockdown MHCC97H and Li-7 cells and their control cells. GAPDH was used as a loading control. **f** The growth curves of xenograft tumors derived from *DDOST*-knockdown MHCC97H cells (*n* = 5) and control cells (*n* = 5). **g** The images (left) and weights (right) of *DDOST*-knockdown xenograft tumors and control tumors. **h** IF staining of DDOST and Ki-67 in the indicated tumor tissues. Scale bar, 100 μm. *P < 0.05, **P < 0.01, ***P < 0.001 (two-way ANOVA for **b** and **f** unpaired two-tailed Student’s *t*-test for **c**, **d** and **g**).

To assess the biological roles of DDOST in HCC cells, we first knocked down *DDOST* in MHCC97H, Li-7 and Huh7 cells using two different siRNAs targeting *DDOST* (si-DDOST-1 and si-DDOST-3), as confirmed by western blotting analysis (Fig. 2a and Supplementary Fig. 3a). Knockdown of *DDOST* significantly suppressed HCC cell proliferation (Fig. 2b) and colony formation (Fig. 2c and Supplementary Fig. 3b), demonstrating its antitransformation effects. Moreover, *DDOST* knockdown caused cell-cycle G_2_/M arrest in MHCC97H and Li-7 cells (Fig. 2d and Supplementary Fig. 3c), accompanied by upregulation of p21^Waf1/Cip1^ and decreased expression of cyclin A1, cyclin B1 and cyclin D1 (Fig. 2e). Moreover, we stably knocked down *DDOST* in MHCC97H cells (Supplementary Fig. 4) and established subcutaneous xenograft tumor models using these cells to explore the tumorigenic potential of DDOST in vivo. The results showed inhibitory effects of *DDOST* knockdown on both in vivo tumor growth and ex vivo tumor volumes and weights (Fig. 2f,g). Furthermore, the levels of Ki-67, one of the proliferation markers, was clearly reduced in *DDOST*-knockdown tumors compared with control tumors (Fig. 2h and Supplementary Fig. 5). Conversely, we ectopically expressed *DDOST* in MHCC97H, Li-7 and Huh7 cells (Supplementary Fig. 6a,b), and demonstrated its protransformation effects on cell proliferation and colony formation assays (Supplementary Fig. 6c,d). The above results further support the pivotal role of DDOST in the progression of HCC.

While STT3A, the catalytic OST subunit, has been well characterized as both the enzymatic core and a druggable target, the STT3A dependence of the role of DDOST in HCC pathogenesis remains unresolved. To comparatively assess their functional contributions, we performed targeted knockdown of *DDOST* and *STT3A* in HCC cell lines. Knockdown of either *DDOST* or *STT3A* significantly suppressed cellular proliferation (Supplementary Fig. 7a) and colony formation capacity (Supplementary Fig. 7b). Notably, knockdown of *DDOST* consistently produced more pronounced phenotypic effects across all assays.

To delineate the mechanistic distinctions between these OST components, we conducted RNA sequencing analysis following *DDOST* or *STT3A* knockdown in MHCC97H cells. Comparative transcriptomic profiling revealed 3,470 DEGs in *DDOST*-knockdown cells versus 1,310 DEGs in *STT3A*-knockdown cells, with 461 overlapping transcriptional changes (Supplementary Fig. 8a,b). Pathway enrichment analysis of coregulated genes identified shared involvement in antiviral defense responses, innate immune regulation, cytokine-mediated signaling pathways and tyrosine metabolism, confirming their cooperative role in OST-mediated glycosylation. More importantly, *DDOST* knockdown specifically downregulated genes involved in fundamental cell cycle processes including mitotic nuclear division, chromosome segregation and DNA replication, suggesting potential OST-independent roles in the regulation of cell proliferation. STT3A-selective effects predominantly involved immune response modulation and fibroblast proliferation regulation, consistent with its established canonical functions in the OST complex (Supplementary Fig. 8c–e). These findings demonstrate that, while DDOST shares some STT3A-dependent functions, it also exerts unique regulatory roles in HCC pathogenesis. The broader phenotypic impact, more extensive transcriptomic remodeling and distinct pathway regulation observed upon *DDOST* depletion make it a promising and mechanistically rational therapeutic target.

**Fig. 3:**
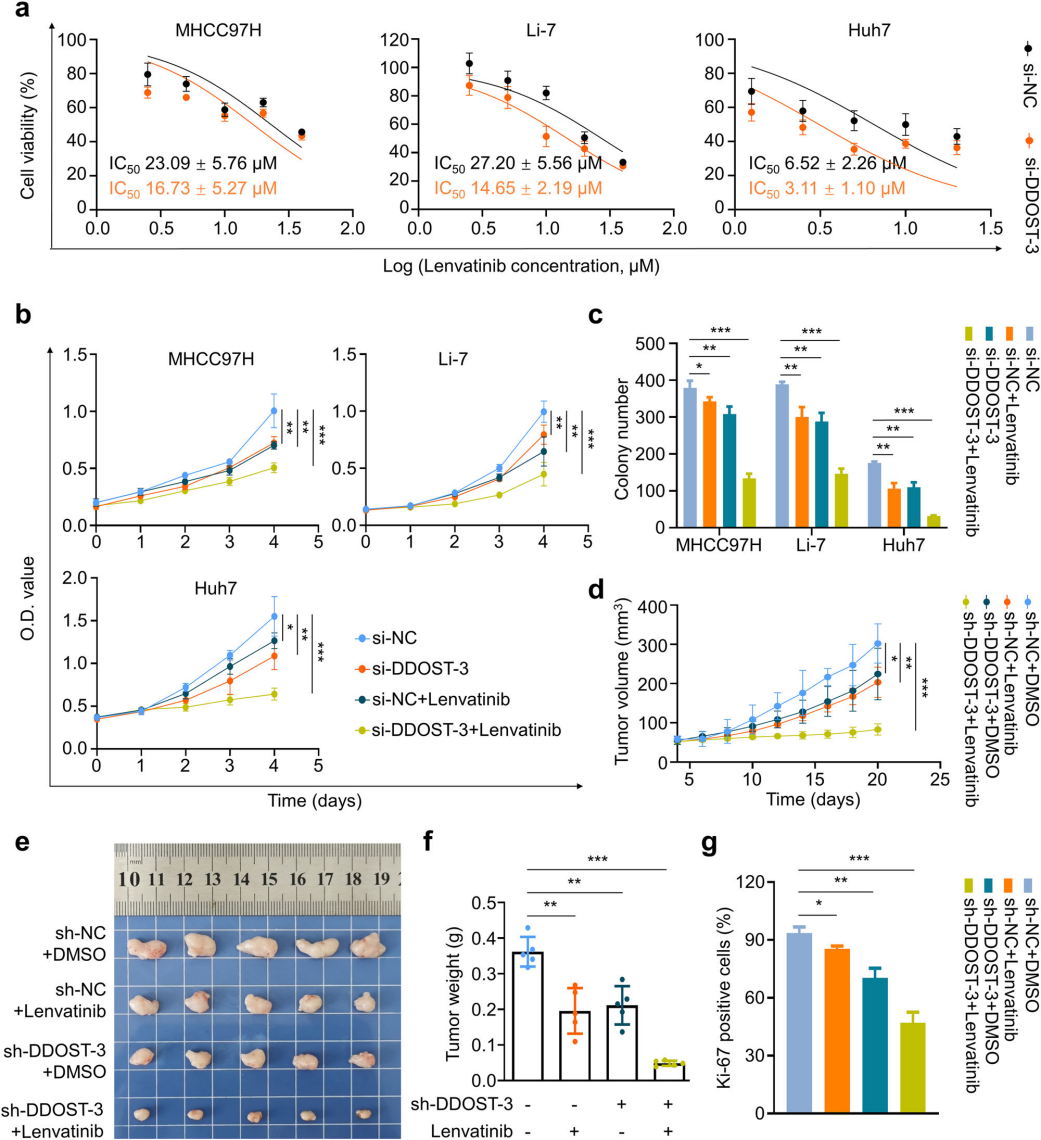
*DDOST* knockdown improves the efficacy of lenvatinib in HCC cells. **a** The IC_50_ values of lenvatinib in *DDOST*-knockdown MHCC97H, Li-7 and Huh7 cells as well as their control cells. **b** *DDOST*-knockdown MHCC97H, Li-7 and Huh7 cells or their control cells were treated with lenvatinib, and MTT assays were then performed to evaluate their effect on cell proliferation. **c** Colony formation of MHCC97H, Li-7 and Huh7 cells with the indicated treatments. **d** The growth curves of the indicated xenograft tumors (*n* = 5 per group). The images (**e**) and weights (**f**) of the indicated tumors. **g** Statistical analysis on IHC staining of Ki-67 in the indicated tumor tissues. Data are expressed as mean ± s.d. *P < 0.05, **P < 0.01, ***P < 0.001 (two-way ANOVA for **b** and **d**, unpaired two-tailed Student’s *t*-test for **c**, **f** and **g**).

Given that *DDOST*/*STT3A* knockdown cosuppresses ERBB2 (a heterodimer of EGFR) signaling (Supplementary Fig. 8c) and aberrant EGFR activation contributes to lenvatinib resistance, we next investigated whether DDOST regulates lenvatinib resistance in HCC cells. Our data showed that *DDOST* knockdown decreased the IC_50_ values from 23.09 ± 5.76 μM to 16.73 ± 5.27 μM in MHCC97H cells, from 27.20 ± 5.56 μM to 14.65 ± 2.19 μM in Li-7 cells and from 6.52 ± 2.26 μM to 3.11 ± 1.10 μM in Huh7 cells (Fig. 3a). Moreover, *DDOST* knockdown enhanced the inhibitory effects of lenvatinib on cell viability in MTT and colony formation assays (Fig. 3b, c and Supplementary Fig. 9). To comparatively analyze the effects of DDOST and STT3A on lenvatinib resistance, we evaluated cellular proliferation and colony formation in HCC cells following *DDOST* or *STT3A* knockdown under lenvatinib treatment. Notably, while both *DDOST* and *STT3A* knockdown sensitized HCC cells to lenvatinib, *DDOST* depletion showed significantly greater sensitization efficacy than *STT3A* knockdown (Supplementary Fig. 10a, b). These data demonstrate that the OST complex regulates lenvatinib resistance in HCC, with DDOST potentially exerting additional antitransformation effects that promote resistance.

Moreover, we gave lenvatinib to nude mice harboring xenograft tumors derived from *DDOST*-knockdown MHCC97H cells and found that *DDOST* knockdown substantially enhanced antitumor efficacy of lenvatinib, as indicated by tumor growth curves (Fig. 3d), tumor volumes (Fig. 3e) and weights (Fig. 3f). IHC staining of Ki-67 further supported the above conclusions (Fig. 3g and Supplementary Fig. 11). Notably, all treatments did not cause a significant effect on body weights of mice (Supplementary Fig. 12).

### DDOST contributes to lenvatinib resistance in HCC by governing the EGFR signaling pathway

**Fig. 4:**
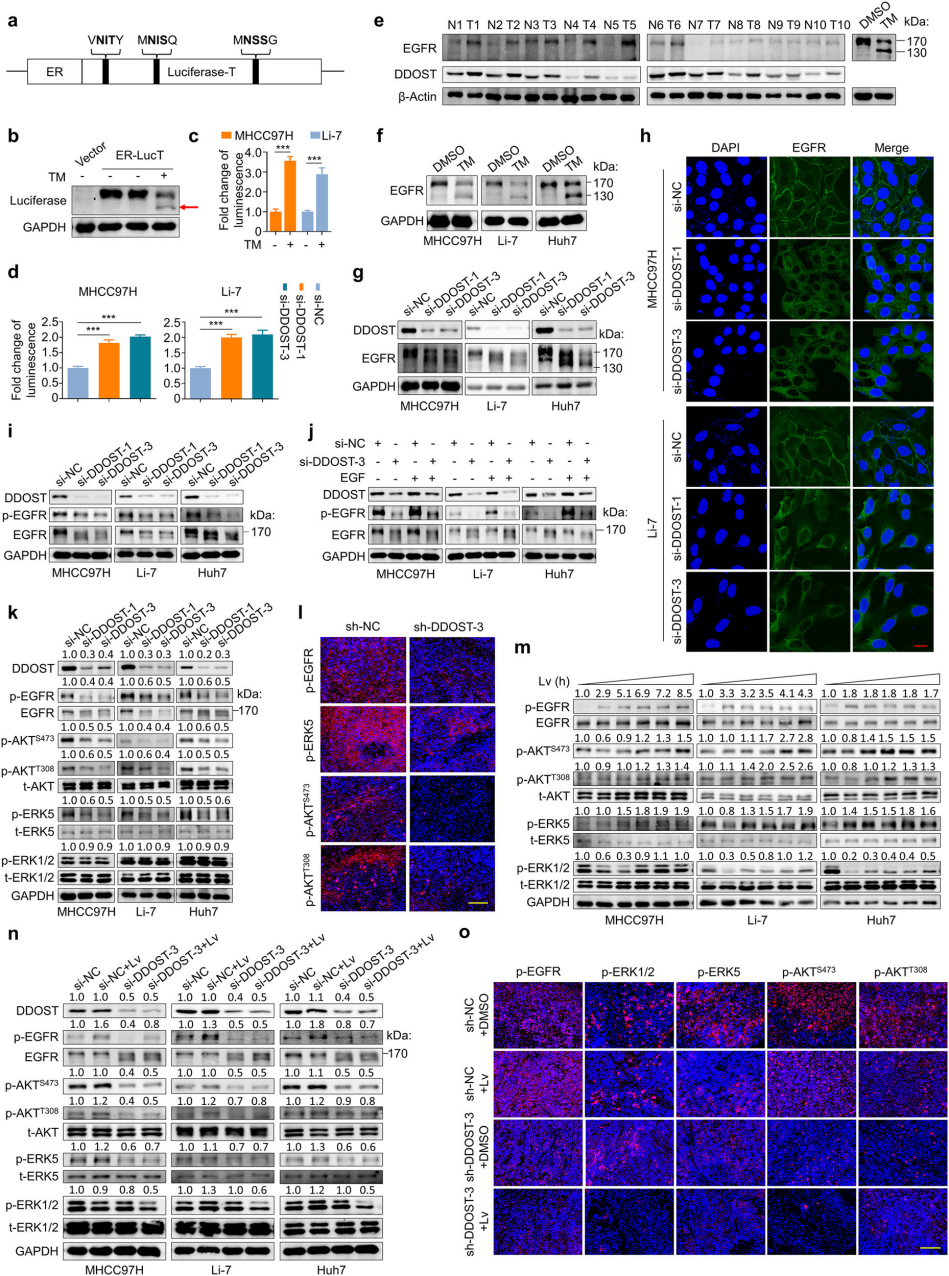
*DDOST* knockdown inhibits N-glycosylation and signaling activity of EGFR in HCC cells. **a** Schematic diagram of the ER-LucT reporter structure. **b** Western blotting analysis showing the expression of N-glycosylated ER-LucT and its response to TM in MHCC97H cells transfected with the ER-LucT reporter plasmid. The red arrow indicates nonglycosylated proteins. GAPDH was used as a loading control. **c** Luminescence assays evaluating the effect of TM on N-glycosylation of ER-LucT fusion protein. **d** Luminescence assays evaluating the effect of *DDOST* knockdown on the N-glysosylation of ER-LucT fusion protein. **e** Western blotting analysis of EGFR and DDOST protein expression in a panel of HCC tissues (T, *n* = 10) and matched noncancerous liver tissues (N, *n* = 10). β-Actin was used as a loading control. Western blotting analysis showing the effects of TM (**f**) and *DDOST* knockdown (**g**) on N-glycosylation of EGFR in MHCC97H, Li-7 and Huh7 cells. GAPDH was used as a loading control. **h** IF staining showing the subcellular distribution of EGFR in MHCC97H and Li-7 cells. Scale bar, 20 μm. **i**, **j** Western blotting analysis showing the effect of *DDOST* knockdown on the phosphorylation of EGFR in MHCC97H, Li-7 and Huh7 cells (**i**), and in response to exogenous EGF stimulation (**j**). GAPDH was used as a loading control. **k** Western blotting analysis showing the effect of *DDOST* knockdown on the phosphorylation of EGFR, AKT, ERK5 and ERK1/2 in *DDOST*-knockdown MHCC97H, Li-7 and Huh7 cells and their control cells. GAPDH was used as a loading control. **l** IF staining of EGFR signaling-related molecules including p-EGFR, p-AKT, p-ERK5 and p-ERK1/2 in DDOST-knockdown tumors and control tumors. Scale bar, 100 μm. **m** Western blotting analysis of EGFR signaling-related molecules in MHCC97H, Li-7 and Huh7 cells with the increase of lenvatinib treatment time (from 0.5 h to 6.0 h). GAPDH was used as a loading control. **n** Western blotting analysis of EGFR signaling-related molecules in MHCC97H, Li-7 and Huh7 cells with the indicated treatments. GAPDH was used as a loading control. **o** Representative IF images of p-EGFR, p-ERK1/2, p-ERK5 and p-AKT in the indicated tumor tissues. Scale bar, 100 μm. Lv, lenvatinib. Data are expressed as mean ± s.d. ***P < 0.001 (unpaired two-tailed Student’s *t*-test for **c** and **d**).

The role of DDOST in the process of N-glycosylation of proteins has been presented in recent studies^45,46^. To confirm the association of the function of DDOST in N-glycosylation modification with its oncogenic role in HCC, we established the ER-LucT reporter system as previously described^47^. In brief, three N-glycosylation sites (NIT, NIS and NSS) were included in an ER-luciferase fusion protein (Fig. 4a). Glycosylation can increase the molecular weight of fusion protein and abolish luciferase activity. The administration of TM, an N-glycosylation inhibitor, successfully restored both molecular weight of fusion protein and luciferase activity in MHCC97H and Li-7 cells (Fig. 4b,c), validating the reliability of this reporter system. Using this system, we found that si-DDOST-1 and si-DDOST-3 significantly increased the luminescence intensity. Specifically, si-DDOST-1 caused a 1.81 ± 0.10-fold increase, while si-DDOST-3 caused a 2.02 ± 0.06-fold increase in MHCC97H cells. In Li-7 cells, si-DDOST-1 caused a 1.99 ± 0.10-fold increase, and si-DDOST-3 caused a 2.10 ± 0.14-fold increase (Fig. 4d). These data demonstrate the crucial role of DDOST in protein N-glycosylation.

Glycans account for approximately 40 kDa of EGFR molecular weight^48^ and are critical in determining its conformation, dimerization and signal transduction^49,50^. Considering the involvement of EGFR in lenvatinib resistance, we speculated whether the enhanced response of HCC cells to lenvatinib following *DDOST* knockdown might be related to the N-glycosylation of EGFR. Western blot analysis revealed that EGFR migrated at ~170 kDa, consistent with its glycosylated form. Notably, both DDOST and glycosylated EGFR were significantly upregulated in HCC tissues (8/10 cases) compared with adjacent normal liver tissues (Fig. 4e). TM treatment caused a reduction of partial EGFR from 170 kDa to 130 kDa in MHCC97H, Li-7 and Huh7 cells, indicating the presence of nonglycosylated EGFR (Fig. 4f). *DDOST* knockdown also caused a decrease in the molecular weight of some EGFR proteins in all three cell lines (Fig. 4g), suggesting that DDOST may be involved in regulating N-glycosylation of EGFR proteins. Nevertheless, *DDOST* knockdown did not affect the mRNA levels of *EGFR* (Supplementary Fig. 13).

Next, IF staining assays were utilized to examine the effect of *DDOST* knockdown on the subcellular distribution of EGFR in MHCC97H and Li-7 cells. The results showed that *DDOST* knockdown led to the loss of EGFR localization on cell membrane, making it relocate to the cytoplasm (Fig. 4h), which was accompanied by a reduction in phosphorylation levels of EGFR (Fig. 4i). In addition, exogenous EGF stimulation did not change the suppressive effect on EGFR activity in *DDOST*-knockdown cells (Fig. 4j). Correspondingly, EGFR downstream effectors, including phosphorylated AKT (p-AKT Ser473 and Thr308) and phosphorylated ERK5 (p-ERK5), was significantly reduced upon *DDOST* knockdown; however, phosphorylation of ERK1/2 remained unchanged (Fig. 4k). Similarly, the levels of p-EGFR, p-AKT (Ser473), p-AKT (Thr308) and p-ERK5 in xenograft tumors were obviously decreased upon *DDOST* knockdown (Fig. 4l and Supplementary Fig. 14). As expected, ectopic expression of *DDOST* increased the phosphorylation levels of EGFR, ERK5 and AKT (Ser473 and Thr308) in MHCC97H, Li-7 and Huh7 cells (Supplementary Fig. 15). These data demonstrate the regulatory role of DDOST in EGFR signaling.

To investigate the role of DDOST-mediated EGFR signaling in lenvatinib resistance, we first assessed the effect of lenvatinib on cell viability in MTT and colony formation assays in the presence of exogenous EGF and confirmed that EGFR activation significantly diminished the antitumor effects of lenvatinib (Supplementary Fig. 16a,b). Next, we examined the activity of EGFR-mediated signaling pathways over time in response to 10 μM lenvatinib. As shown in Fig. 4m, the levels of phosphorylated ERK1/2 were significantly inhibited at the early stage of lenvatinib treatment but subsequently rebounded, indicating a shift from sensitivity to resistance. Besides, the phosphorylation levels of EGFR, ERK5 and AKT (Ser473 and Thr308) were progressively increased during lenvatinib treatment, suggesting that the abnormal activation of EGFR signaling may contribute to lenvatinib resistance. Then, we also assessed the impact of *DDOST* knockdown on the EGFR activation and downstream signaling when HCC cells were exposed to lenvatinib for 6 h. As shown in Fig. 4n, *DDOST* knockdown induced the deglycosylation of EGFR and prevented the activation of EGFR, AKT and ERK5, while inhibiting the rebound of p-ERK1/2 mediated by lenvatinib. In addition, immunofluorescence staining of several effectors (p-EGFR, p-AKT, p-ERK1/2 and p-ERK5) in *DDOST*-knockdown and control xenograft tumors treated with lenvatinib further supported these conclusions (Fig. 4o and Supplementary Fig. 17). To compare between OST-dependent and OST-independent mechanisms in *DDOST* knockdown effects, we examined changes in EGFR and its downstream signaling molecules under lenvatinib treatment after *DDOST* or *STT3A* knockdown. Both knockdowns inhibited the activation of EGFR, AKT, ERK5 and ERK1/2, but *DDOST* depletion showed significantly stronger suppression of EGFR and all three effector molecules compared with *STT3A* knockdown (Supplementary Fig. 18). Collectively, our data indicate that DDOST governs the EGFR signaling pathway to induce the resistance of HCC cells to lenvatinib.

### *DDOST* knockdown hinders the glycosylation and membrane localization of PD-L1

**Fig. 5:**
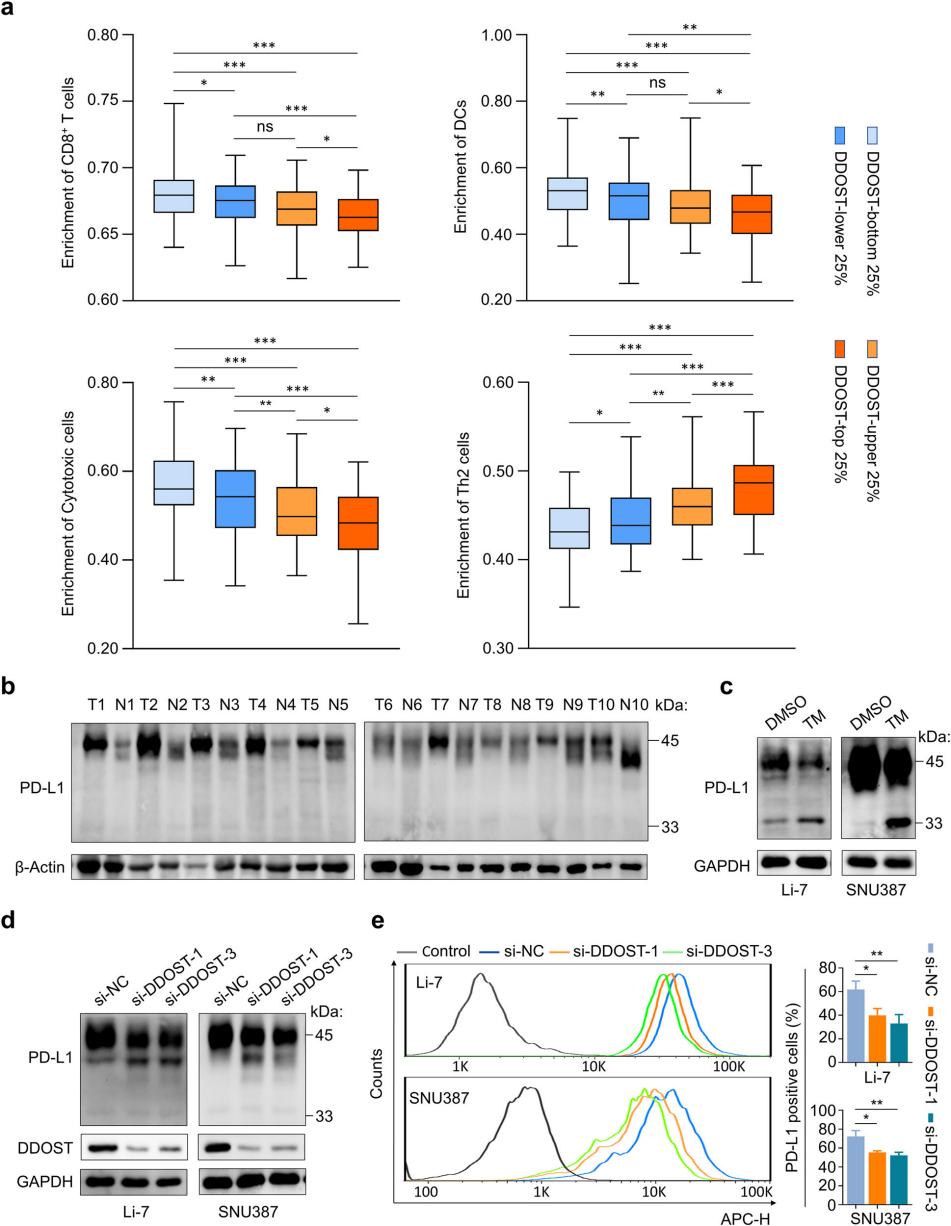
*DDOST* knockdown impedes the glycosylation of PD-L1 and its membrane localization. **a** The infiltration levels of immune cells across quartile groups of *DDOST* expression in HCC tissues (*n* = 85 per group), with each quartile representing 25% of the TCGA cohort ranked by *DDOST* expression levels (data from TCGA database). **b** Western blotting analysis showing PD-L1 expression in HCC tissues (T, *n* = 10) and matched noncancerous liver tissues (N, *n* = 10). β-Actin was used as a loading control. **c**, **d** Western blotting analysis showing the effects of TM (**c**) and *DDOST* knockdown (**d**) on N-glycosylation of PD-L1 in Li-7 and SNU387 cells. GAPDH was used as a loading control. **e** Flow cytometry evaluating membrane localization of PD-L1 in DDOST-knockdown Li-7 and SNU387 cells and their control cells (left), and the statistical results (right). Data are expressed as mean ± s.d. *P < 0.05, **P < 0.01, ***P < 0.001 (unpaired two-tailed Student’s *t*-test for **a** and **e**).

N-glycosylation of PD-L1 is essential for its interaction with PD-1^51^. We thus hypothesize that elevated *DDOST* expression in HCCs may affect the immune-suppressive function of PD-L1. By analyzing the TCGA database, we observed a stepwise association between *DDOST* expression levels and immune cell composition in HCC. Increasing *DDOST* expression correlated with significantly reduced enrichment of antitumor immune cells (CD8⁺ T cells, cytotoxic cells and dendritic cells), whereas immunosuppressive Th2 cells displayed progressively higher enrichment in the high-DDOST groups (Fig. 5a). These findings suggest that DDOST may be related to the immune escape of HCC.

We next examined the levels of glycosylated PD-L1 in ten pairs of HCC tissues and noncancerous liver tissues by western blotting analysis, and found that PD-L1 was highly expressed in most cancer tissues (7/10) and appeared as fully glycosylated proteins (45 kDa) compared with noncancerous liver tissues (Fig. 5b). Moreover, we treated Li-7 and SNU387 cells with N-glycosylation inhibitor TM, and we observed that PD-L1 existed both as fully glycosylated proteins (Fig. 5c and Supplementary Fig. 19a) and as the nonglycosylated form (33 kDa), which was substantially increased upon TM treatment (Fig. 5c). Also, *DDOST* knockdown decreased the levels of fully glycosylated PD-L1 and induced incomplete glycosylation of PD-L1 in Li-7 and SNU387 cells (Fig. 5d). However, *DDOST* knockdown had no impact on the transcription of *PD-L1* (Supplementary Fig. 19b). In addition, flow cytometry analysis demonstrated that *DDOST* knockdown clearly decreased the levels of PD-L1 on the surface of Li-7 and SNU387 cells (Fig. 5e). These findings suggest that DDOST-mediated N-glycosylation is crucial for the membrane localization of PD-L1 in HCC cells.

### NGI-1 enhances the efficacy of lenvatinib in HCC by suppressing EGFR glycosylation

**Fig. 6:**
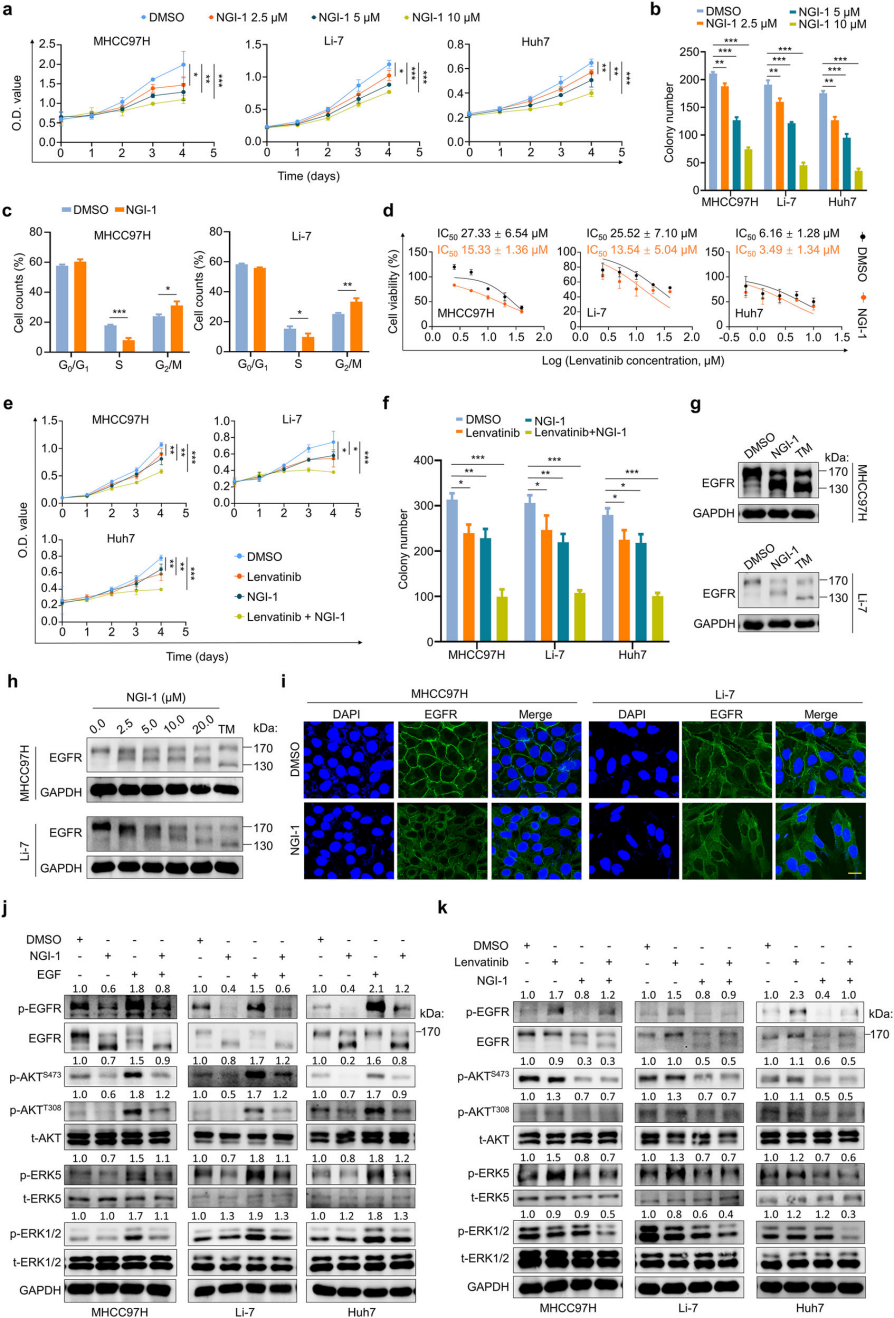
Antitumor effects of NGI-1 and its sensitization to lenvatinib in HCC cells. **a** MTT assays evaluating the inhibitory effect of NGI-1 on the proliferation of MHCC97H, Li-7 and Huh7 cells. **b** Colony formation ability of MHCC97H, Li-7 and Huh7 cells treated with different doses of NGI-1. **c** Cell cycle distribution in MHCC97H and Li-7 treated with DMSO or NGI-1. **d** The IC_50_ values of lenvatinib in MHCC97H, Li-7 and Huh7 cells pretreated with 5 μM NGI-1 for 24 h. **e** MTT assays evaluating the proliferation of MHCC97H, Li-7 and Huh7 cells treated with NGI-1 and lenvatinib, individually or in combination. **f** Colony formation ability of MHCC97H, Li-7 and Huh7 cells treated with NGI-1 and lenvatinib, individually or in combination. **g** MHCC97H and Li-7 cells were treated with 10 μM NGI-1 for 24 h, and western blotting analysis was then used to determine its effect on EGFR glycosylation. GAPDH was used as a loading control. **h** MHCC97H and Li-7 cells were treated with different concentrations of NGI-1 for 24 h, and western blotting analysis was then performed to determine its effect on EGFR glycosylation. GAPDH was used as a loading control. **i**, IF staining showing the subcellular distribution of EGFR in MHCC97H and Li-7 cells treated with DMSO or 10 μM NGI-1 for 24 h. Scale bar, 20 μm. **j** Western blotting analysis showing the effect of NGI-1 on the levels of EGFR signaling-related molecules in MHCC97H, Li-7 and Huh7 cells in presence or absence of EGF stimulation. GAPDH was used as a loading control. **k** Western blotting analysis of EGFR signaling-related molecules in MHCC97H, Li-7 and Huh7 cells with the indicated treatments. GAPDH was used as a loading control. Data are expressed as mean ± s.d. *P < 0.05, **P < 0.01, ***P < 0.001 (two-way ANOVA for **a** and **e** unpaired two-tailed Student’s *t*-test for **b**, **c** and **f**).

Our data confirm that N-glycosylation is required for EGFR activity. NGI-1, a small-molecule inhibitor targeting catalytic subunits of OST (STT3A and STT3B), substantially suppresses cellular N-glycosylation^52^. This was demonstrated in HCC cells using ER-LucT reporter assays (Supplementary Fig. 20). Next, we treated MHCC97H, Li-7 and Huh7 cells with NGI-1 and found that NGI-1 exhibited antitransformation effects in a dose-dependent manner on both MTT (Fig. 6a) and colony formation assays (Fig. 6b and Supplementary Fig. 21). Moreover, NGI-1 induced G_2_/M phase cell-cycle arrest after a 24-h treatment (Fig. 6c and Supplementary Fig. 22), accompanied by increased p21^Waf1/Cip1^ expression and decreased levels of cyclin D1, cyclin A1 and cyclin B1 in MHCC97H and Li-7 cells (Supplementary Fig. 23). NGI-1 had a similar effect with *DDOST* knockdown, increasing the sensitivity to lenvatinib in HCC cells, as indicated by reduced IC_50_ values in MHCC97H (from 27.33 ± 6.54 μM to 15.33 ± 1.36 μM), Li-7 (from 25.52 ± 7.10 μM to 13.54 ± 5.04 μM) and Huh7 cells (from 6.16 ± 1.28 μM to 3.49 ± 1.34 μM) (Fig. 6d). As expected, NGI-1 enhanced the antitransformation effects of lenvatinib in MTT and colony formation assays (Fig. 6e,f and Supplementary Fig. 24).

To determine whether antitumor effects of NGI-1 in HCC depend on the glycosylation of EGFR, we assessed the changes in the molecular weight of EGFR proteins in HCC cells exposed to NGI-1. TM was used as a positive control. The results showed that NGI-1 partially inhibited EGFR glycosylation, leading to a decrease in its molecular weight (Fig. 6g). With the increase in NGI-1 doses, low molecular weight EGFR showed a dose-dependent increase (Fig. 6h), but its mRNA levels were unchanged upon NGI-1 treatment (Supplementary Fig. 25). In addition, NGI-1 significantly disrupted the membrane localization of EGFR in both MHCC97H and Li-7 cells (Fig. 6i). Western blotting analysis also demonstrated that NGI-1 substantially reduced the phosphorylation levels of EGFR, AKT (Ser473 and Thr308) and ERK5 in HCC cells, regardless of whether these cells were treated with EGF (Fig. 6j). In line with the enhanced sensitivity of lenvatinib, NGI-1 also suppressed the rebound of p-ERK1/2 mediated by lenvatinib treatment (Fig. 6k). The above findings suggest that NGI-1 inhibits N-glycosylation of EGFR to blocks its signaling activity, thus alleviating EGFR-mediated resistance to lenvatinib in HCC cells.

### The preparation and antitumor effects of NGI-1-NPs

**Fig. 7:**
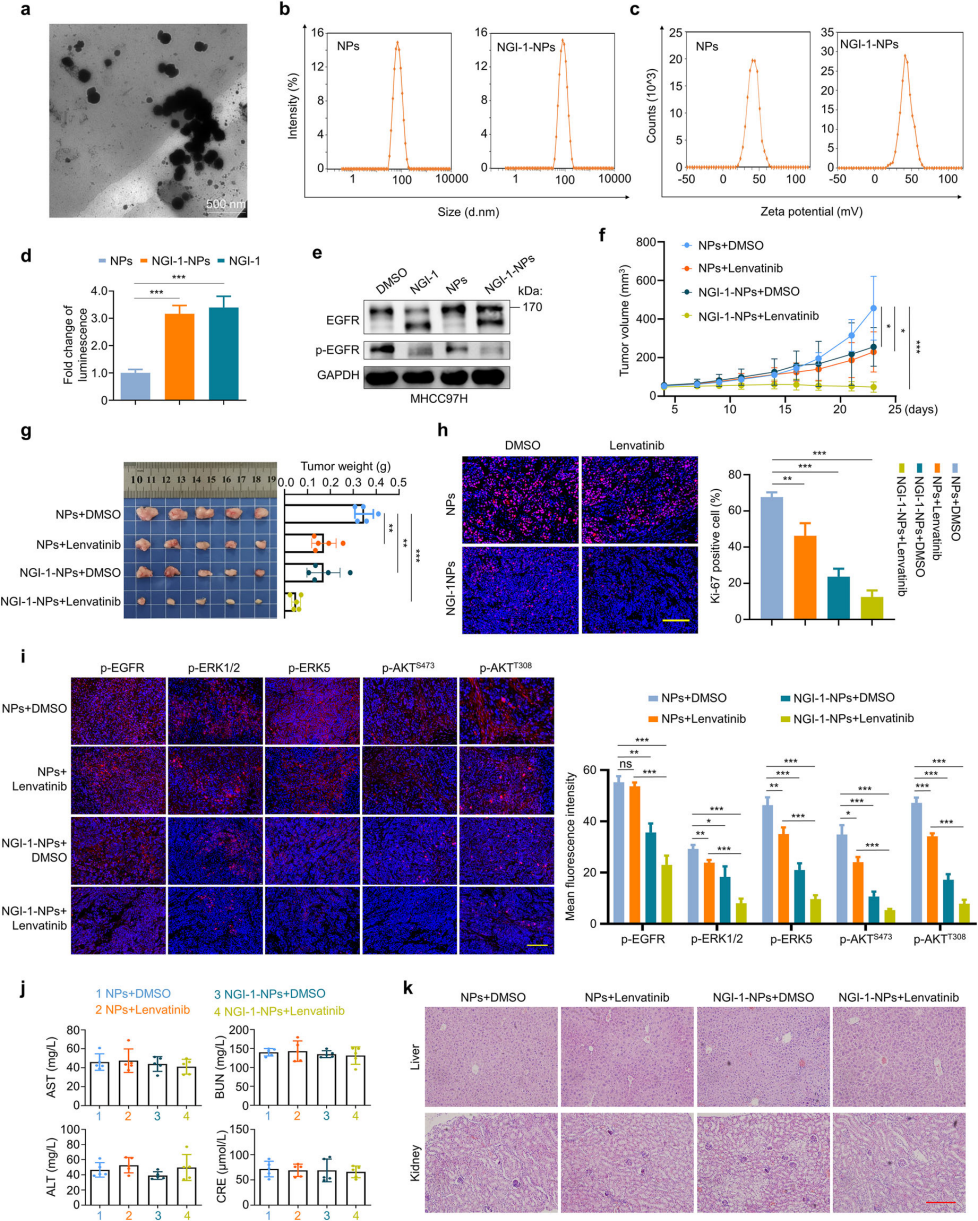
The characterizations of NGI-1-NPs and their antitumor effects in HCC cells. **a** Representative TEM image of NGI-1-NPs. Scale bar, 500 nm. **b** Particle sizes of NGI-1-NPs and NPs measured by dynamic light scattering. **c** Zeta potentials of NGI-1-NPs and NPs. **d** Luminescence assay evaluating the effect of NGI-1-NPs on N-glycosylation of E-lucT fusion protein. NGI-1 was used as a positive control. **e** Western blotting analysis showing the effect of NGI-1-NPs on N-glycosylation and phosphorylation of EGFR in MHCC97H cells. **f** The growth curves of xenograft tumors in different groups (*n* = 5 per group). **g** The images (left) and weights (right) of xenograft tumors with the indicated treatments. **h** IF staining of Ki-67 in the indicated tumor tissues. Left: representative images of IF. Right: the statistical results. Scale bar, 100 μm. **i**, IF staining of p-EGFR, p-ERK5 and p-AKT (S473 and T308) in tumor tissues with the indicated treatments. Left: the representative images of IF. Right: the statistical results. Scale bar, 100 μm. **j** The levels of AST, ALT, CRE and BUN in mice with the indicated treatments. **k** Representative H&E staining images of liver and kidney tissues in mice with the indicated treatments. Scale bar, 100 μm. Data are expressed as mean ± s.d. *P < 0.05, **P < 0.01, ***P < 0.001 (unpaired two-tailed Student’s *t*-test for **d** and **g**–**i**, two-way ANOVA for **f**).

To overcome the challenge associated with the poor solubility of NGI-1, which greatly limits its application, we explored the use of nanoparticles (NPs) to improve its bioavailability. Considering the advantages of NPs in delivering poorly soluble drugs^53,54^, we used PEG–PLGA amphiphilic block copolymers and the nanoprecipitation technique to formulate NGI-1-NPs and control NPs. Transmission electron microscopy (TEM) confirmed that NGI-1-NPs were spherical with uniform size and well-defined edges (Fig. 7a). Dynamic light scattering analysis indicated that the average particle sizes of NGI-1-NPs and NPs were 79.46 ± 1.71 nm and 69.30 ± 2.32 nm, respectively (Fig. 7b). The corresponding zeta potentials were measured as 43.10 ± 0.90 mV and 41.90 ± 0.15 mV (Fig. 7c). High-performance liquid chromatography analysis showed the encapsulation efficiency of NGI-1-NPs was 50.06 ± 3.15%. Next, we validated the inhibitory effect of NGI-1-NPs on protein N-glycosylation using ER-LucT reporter assays (Fig. 7d). Western blotting analysis also demonstrated that NGI-1-NPs effectively suppressed EGFR glycosylation (Fig. 7e).

To determine the antitumor efficacy of NGI-1-NPs, we established a xenograft mouse model through subcutaneously inoculating MHCC97H cells. Then, the mice were randomly grouped and received NPs, lenvatinib, NGI-1-NPs or a combination of NGI-1-NPs and lenvatinib. The results showed that NGI-1-NP treatment alone substantially inhibited both in vivo tumor growth curves and ex vivo tumor weights, and these effects were more significant when combined with lenvatinib (Fig. 7f,g). IF staining of Ki-67 in tumor sections further supported this conclusion (Fig. 7h). Moreover, IF staining indicated that combined treatment with lenvatinib and NGI-1-NPs clearly decreased the levels of p-EGFR and its downstream effectors compared with monotherapy (Fig. 7i). Despite a slight and transient loss in body weights of mice in the combination therapy group (Supplementary Fig. 26), there was no significant effect on liver and kidney function (Fig. 7j). H&E staining of liver and kidney sections further supported its biosafety (Fig. 7k).

Given the potential impact of OST inhibitors on broad-spectrum protein glycosylation, we additionally assessed the systemic toxicity of NGI-1 by analyzing hematological parameters and histopathology of major organs. NGI-1-NP treatment showed no significant effects on peripheral blood counts including erythrocytes, hemoglobin, leukocytes, platelets and neutrophil/lymphocyte ratios (Supplementary Fig. 27a), bone marrow nucleated cell counts (Supplementary Fig. 27b,c) or myeloid-to-erythroid (M/E) ratios (4.5:1 in NP controls versus 3.5:1 in NGI-1-NP-treated mice). Histological evaluation of lung, spleen and heart tissues further confirmed the safety profile of NGI-1-NPs (Supplementary Fig. 27d). These results suggest that NGI-1-NPs safely enhance NGI-1 bioavailability and synergize with lenvatinib in HCC treatment.

### NGI-1-NPs enhances the efficacy of immunotherapy

**Fig. 8:**
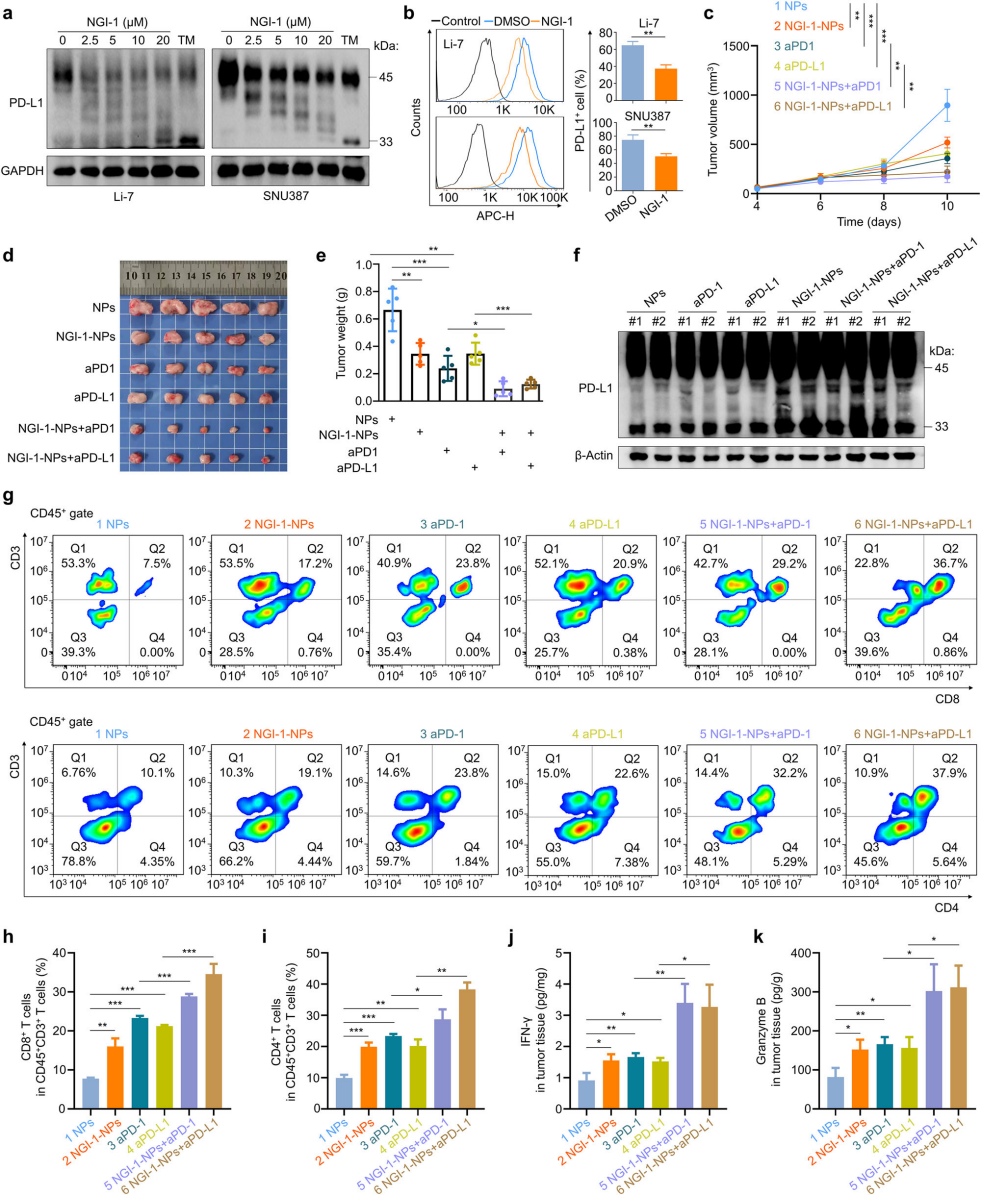
NGI-1-NPs block N-glycosylation of PD-L1 and enhance the efficacy of immunotherapy in HCC. **a** Western blotting assay showing the dose-dependent inhibitory effect of NGI-1 on N-glycosylation of PD-L1 in Li-7 and SNU387 cells. GAPDH was used as a loading control. **b** Flow cytometry evaluating the membrane localization of PD-L1 in Li-7 and SNU387 cells treated with DMSO or 10 μM NGI-1 for 24 h. **c** Growth curve of H22 cell-derived subcutaneous tumors in the indicated groups. **d**, **e** The images (**d**) and weights (**e**) of the indicated tumors. **f** Western blotting assay evaluating the expression and N-glycosylation of PD-L1 in the indicated tumors. β-Actin was used as a loading control. **g**–**i**, Flow cytometry assessing the infiltration of CD8^+^ T cells (top) and CD4^+^ T cells (bottom) in the indicated tumors (**g**), and the statistical results (**h** and **i**). **j**, **k** The levels of IFN-γ (**j**) and Granzyme B (**k**) in these tumors. Data are expressed as mean ± s.d. *P < 0.05, **P < 0.01, ***P < 0.001 (unpaired two-tailed Student’s *t*-test for **b**, **e** and **h**–**k**, two-way ANOVA for **c**).

Our data confirm that the surface expression of PD-L1 can be modulated by DDOST-mediated N-glycosylation. Thus, we hypothesize that NGI-1 may disrupt the immunosuppressive function of PD-L1, subsequently enhancing the efficacy of immunotherapy. First, we demonstrated that NGI-1 dose-dependently inhibited the levels of N-glycosylated PD-L1 in Li-7 and SNU387 cells but did not change its mRNA levels (Fig. 8a and Supplementary Fig. 28). Moreover, our data also showed that NGI-1 substantially diminished the membrane expression of PD-L1 in Li-7 and SNU387 cells, as determined by flow cytometry analysis (Fig. 8b). As expected, NGI-1-NPs effectively induced incomplete glycosylation of PD-L1 in both human and mouse HCC cell lines (Supplementary Fig. 29). Next, we established a mouse model bearing H22 cell-derived subcutaneous tumors and administered these mice with NGI-1-NPs individually or combined with aPD-1 or aPD-L1. Our data showed that monotherapy by NGI-1-NPs, aPD-1 or aPD-L1 effectively delayed tumor growth, while the combined treatment yielded more favorable therapeutic outcomes (Fig. 8c–e). This was also indicated by IHC staining of Ki-67 in these tumors (Supplementary Fig. 30). Besides, western botting analysis showed that NGI-1-NPs effectively hindered the N-glycosylation of PD-L1 in tumor tissues (Fig. 8f).

Next, we performed flow cytometry to quantify T cells in the above tumors. As shown in Fig. 8g–i, NGI-1-NP, aPD-1 or aPD-L1 monotherapy significantly increased the proportion of T cell infiltration in tumors (CD8^+^ T: from 7.5% to 17.2% in the NGI-1-NPs group, 23.8% in the aPD-1 group and 20.9% in the aPD-L1 group; CD4^+^ T: from 10.1% to 19.1% in the NGI-1-NPs group, 23.8% in the aPD-1 group and 22.6% in the aPD-L1 group). As expected, NGI-1-NP treatment combined with anti-PD-1 or anti-PD-L1 resulted in greater T cell infiltration compared with monotherapy. Moreover, the levels of IFN-γ and granzyme B in tumors were greatly elevated by the combined therapies compared with monotherapy (Fig. 8j,k). These data indicate that NGI-1 efficiently enhances T cell infiltration and antitumor activity, thereby markedly improving the response to anti-PD-1 or anti-PD-L1 therapy in HCC.

## Discussion

Aberrant expression of tumor-associated genes frequently results in the deregulation of cell membrane proteins, which are crucial in cancer development and progression^55^. N-glycosylation of proteins is a critical posttranslational modification for the distribution and function of membrane proteins^56^, hence affecting oncogenic signaling pathways^57^. Aberrant glycoprotein expression and changes in N-glycosylation patterns frequently contribute to the carcinogenesis and cancer progression^58^. N-glycosylation involves the transfer of LLO to nascent peptides in the ER, a reaction catalyzed by OST^59^. OST in mammalian cells comprises one of the catalytic subunits and several noncatalytic subunits, including DDOST^60^. Given the critical role of DDOST in OST activity and N-glycosylation, we hypothesize that DDOST may exert an oncogenic effect in human cancers. Previous studies indicated that STT3A and RPN2 promoted tumor progression by maintaining N-glycosylation of key glycoproteins^25,44,61^. DDOST has been demonstrated to be upregulated in various cancer, including bladder, breast and colorectal cancer^33,35,62^, and to be associated with immune infiltration and poor prognosis in HCC^34^. This study confirmed the increase of *DDOST* expression in HCC, which was involved in unsatisfied survival. Moreover, knockdown of *DDOST* in HCC cells led to cell cycle arrest, inhibited cell viability and improved efficacy of lenvatinib in HCC cells, therefore confirming its oncogenic role in HCC. Notably, *DDOST* knockdown exhibited more potent tumor-suppressive effects and broader biological regulatory functions than STT3A inhibition. Its OST complex-independent roles appear to focus on fundamental cell cycle-related processes, as indicated by G_2_/M phase arrest in *DDOST*-depleted cells. This suggests that DDOST may participate in mitotic nuclear division, chromosome segregation and DNA replication independently of canonical OST functions, meriting thorough investigation in future studies.

N-glycans facilitate ligand–receptor interactions, oncogenic signal transduction, cell–cell interactions, cell–matrix interactions and tumor immune evasion^57^. Altered glycosylation can disrupt the activity of receptor tyrosine kinases (RTKs)^63^, such as EGFR, which is highly expressed in HCC. EGFR activation promotes tumor progression and metastasis, and contributes to resistance against therapeutics such as lenvatinib^64^. As demonstrated in this study, the activation of EGFR significantly compromised the efficacy of lenvatinib. It is well documented that N-glycosylation is requisite for EGFR function^48^. Consistent with this, *DDOST* knockdown inhibited EGFR N-glycosylation and further improved the response of HCC cells to lenvatinib. NGI-1, an OST inhibitor targeting both STT3A and STT3B, has been shown to exert antitumor effects in lung cancer by inhibiting the N-glycosylation of EGFR^42^. In the present study, NGI-1, similar to *DDOST* knockdown, inhibited the downstream signaling pathways of EGFR by decreasing its N-glycosylation, thus enhancing the sensitivity of HCC cells to lenvatinib. Moreover, to increase the bioavailability of NGI-1, we constructed NGI-1-NPs and confirmed their in vitro and in vivo antitumor activities in HCC cells.

Our findings indicated that, although lenvatinib initially inhibited ERK1/2 activity in HCC cells, a subsequent rebound in ERK1/2 activity may contribute to the emergence of lenvatinib resistance. Previous studies have shown that tumor cells can reinstate ERK1/2 activity to develop resistance in response to TKIs by either releasing negative feedback mechanisms or activating alternative pathways^65–67^. Lenvatinib targets receptor tyrosine kinases including VEGFR, FGFR, PDGFR and RET, thereby inhibiting the downstream MAPK–ERK1/2 pathway. This subsequently abolishes ERK1/2-mediated suppression of nonlenvatinib targets such as EGFR, establishing compensatory activation mechanisms—a phenomenon that has been repeatedly demonstrated in multiple studies^10,65,68^. Furthermore, the combination of lenvatinib with MEK inhibitors has been shown to augment the antitumor effects of lenvatinib, hence reinforcing the critical involvement of the ERK1/2 signaling pathway in lenvatinib resistance^66^. These observations suggest that combination therapy targeting the ERK1/2 signaling pathway may effectively overcome lenvatinib resistance and improve its therapeutic efficacy. AKT, ERK5 and ERK1/2 constitute the classical effector molecules of the EGFR pathway. Our study comprehensively demonstrates their dynamic alteration during lenvatinib treatment, providing evidence for their roles in lenvatinib resistance. The subsequent findings manifested the regulatory effects of DDOST and OST inhibitor on these signaling nodes. Thus, disruption of N-glycosylation of EGFR by *DDOST* knockdown or NGI-1 treatment, combined with multitargeted inhibition of lenvatinib, effectively attenuated compensatory activation mechanisms and subsequently attenuated lenvatinib resistance.

Emerging evidence suggests that DDOST and its complex may contribute to lenvatinib resistance by regulating the glycosylation of multiple oncogenic substrates. For example, DDOST-mediated glycosylation pathways have been implicated in stabilizing cathepsin D^69^, modifying immune checkpoint ligands (including B7-H4 and HHLA2^70,71^) and facilitating LAMP2 maturation^72^. These processes have been demonstrated to be linked to tumor invasion, immune evasion and stress adaptation. Although the above mechanisms require further validation in the context of lenvatinib resistance, these findings position DDOST-mediated glycosylation as a potential hub coordinating multiple therapeutic resistance pathways, highlighting its possible utility as an anticancer target. However, pharmaceutical targeting of DDOST necessitates rigorous safety evaluation. Murine genetic studies have demonstrated that homozygous *Ddost* ablation results in embryonic lethality, while heterozygous deletion elicits a spectrum of somatic abnormalities, including adrenal hypoplasia, splenic atrophy, lymphadenopathy and impaired erythropoiesis, indicating the indispensable role of DDOST in mammalian development and homeostasis^73,74^. Although the consequences of *DDOST* ablation in mature organisms remain unclear, potential on-target toxicities from its pharmacological inhibition warrant careful evaluation. Future studies should focus on: (1) evaluating the toxicity associated with DDOST inhibition in the adult models and (2) developing tumor-targeted approaches (for example, antibody–drug conjugates or activatable prodrugs) to improve specificity and reduce systemic effects.

N-glycosylation is essential for maintaining PD-L1 protein stability and its high affinity for PD-1, a key mechanism that allows cancer cells to evade immune surveillance^51^. High *DDOST* expression has been indicated to involve increased Th2 cells and decreased infiltration of cytotoxic cells in HCC tissues^34^. Our findings indicated that DDOST suppressed T cell cytotoxicity by preserving the N-glycosylation and plasma membrane localization of PD-L1. This underscores that DDOST not only promotes tumor progression but also serves as a key modulator of the tumor immune microenvironment. Upon knockdown of *DDOST* or treatment of HCC cells with NGI-1, PD-L1 exhibited a hypoglycosylated state, similar to the effect of metformin on PD-L1^22^. Inhibitors targeting N-glycosylation modification of PD-L1 have been consistently reported to inhibit tumor progression and improve the efficacy of immunotherapy^23,24^, as supported by our data showing that NGI-1-NPs improved the efficacy of aPD-1 or aPD-L1 by increasing T cell infiltration in tumors.

**Fig. 9:**
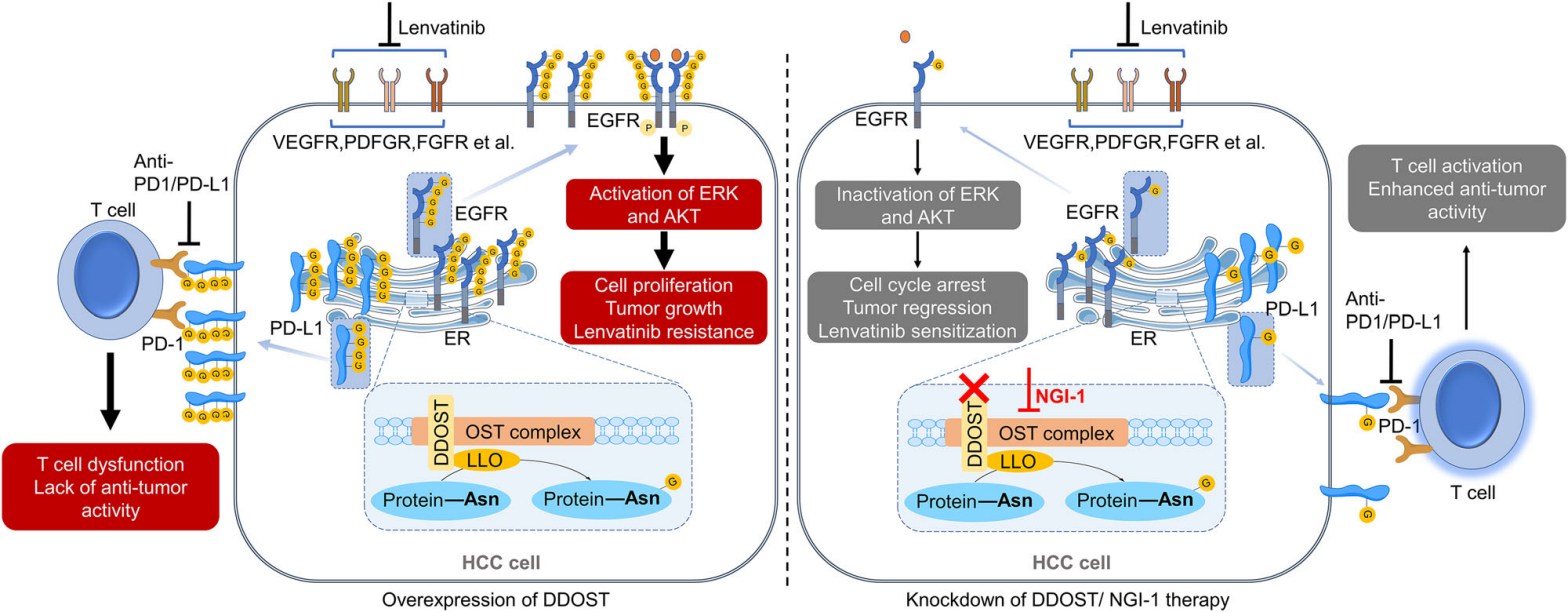
A schematic model illustrating how DDOST-mediated protein glycosylation affects the efficacy of lenvatinib and immunotherapy in HCC. DDOST, when highly expressed in HCC cells, enhanced N-glycosylation of EGFR and PD-L1. On the one hand, it can promote malignant behaviors of HCC cells and their resistance to lenvatinib by activating the downstream signaling pathways of EGFR. On the other hand, it inhibits the cytotoxic effect of T cells by maintaining the membrane localization of PD-L1, decreasing the efficacy of immunotherapy. Thus, knockdown of *DDOST* or treatment with NGI-1 improves the efficacy of lenvatinib and immunotherapy in HCC by blocking N-glycosylation of EGFR and PD-L1.

In summary, our data demonstrated that DDOST, a key noncatalytic subunit of OST, played an oncogenic role in HCC and decreased the response to lenvatinib and immunotherapy by promoting N-glycosylation of EGFR and PD-L1 (Fig. 9). Knockdown of *DDOST* or treatment with NGI-1 hindered the N-glycosylation of EGFR, obstructing the mechanism by which cancer cells compensate for ERK1/2 activity via EGFR, ultimately leading to lenvatinib resistance. The above treatments also decreased the levels of N-glycosylated PD-L1, alleviating the immunosuppressive tumor microenvironment to inhibit HCC growth and improve the efficacy of PD1/PD-L1 inhibitors. Thus, this study provides new evidence involving the mechanisms of drug resistance in HCC and suggests a viable combinatorial therapeutic strategy for HCC.

## Supplementary information

Supplementary Information

## Study approval

All animal experiments were approved by the Laboratory Animal Center of Xi’an Jiaotong University (approval number XJTUAE2024-766) and conducted on the basis of the institutional guidelines. In addition, this study was approved by the Institutional Review Board, Human Ethics Committee of the First Affiliated Hospital of Xi’an Jiaotong University (approval number G-63).
