# Supplementary material for: Targeting DDOST improves the efficacy of lenvatinib and immunotherapy in hepatocellular carcinoma
Source: Exp Mol Med. 2025 Dec 19;57(12):2869–85. doi: 10.1038/s12276-025-01597-9 (PMC12800229; doi:10.1038/s12276-025-01597-9)
Supplement: Supplementary file 1 — Supplementary Information [file 12276_2025_1597_MOESM1_ESM.pdf]

Supplementary Figures

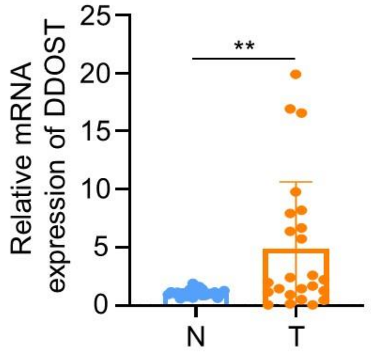

Supplementary Fig. 1 The mRNA expression of *DDOST* in HCC tissues (T, n = 24) and matched non-cancerous liver tissues (N, n = 24). *β-Actin* was used as a normalized control. Data were expressed as mean ± SD. **, $P < 0.01$ (unpaired two-tailed Student's t-test).

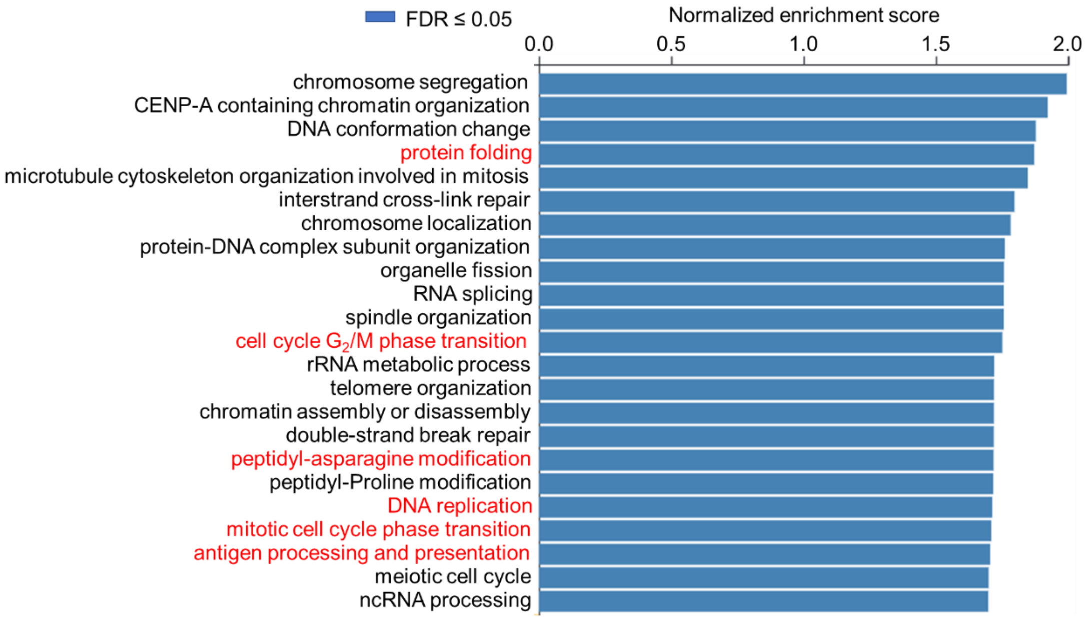

Supplementary Fig. 2 GO enrichment analysis of DDOST.

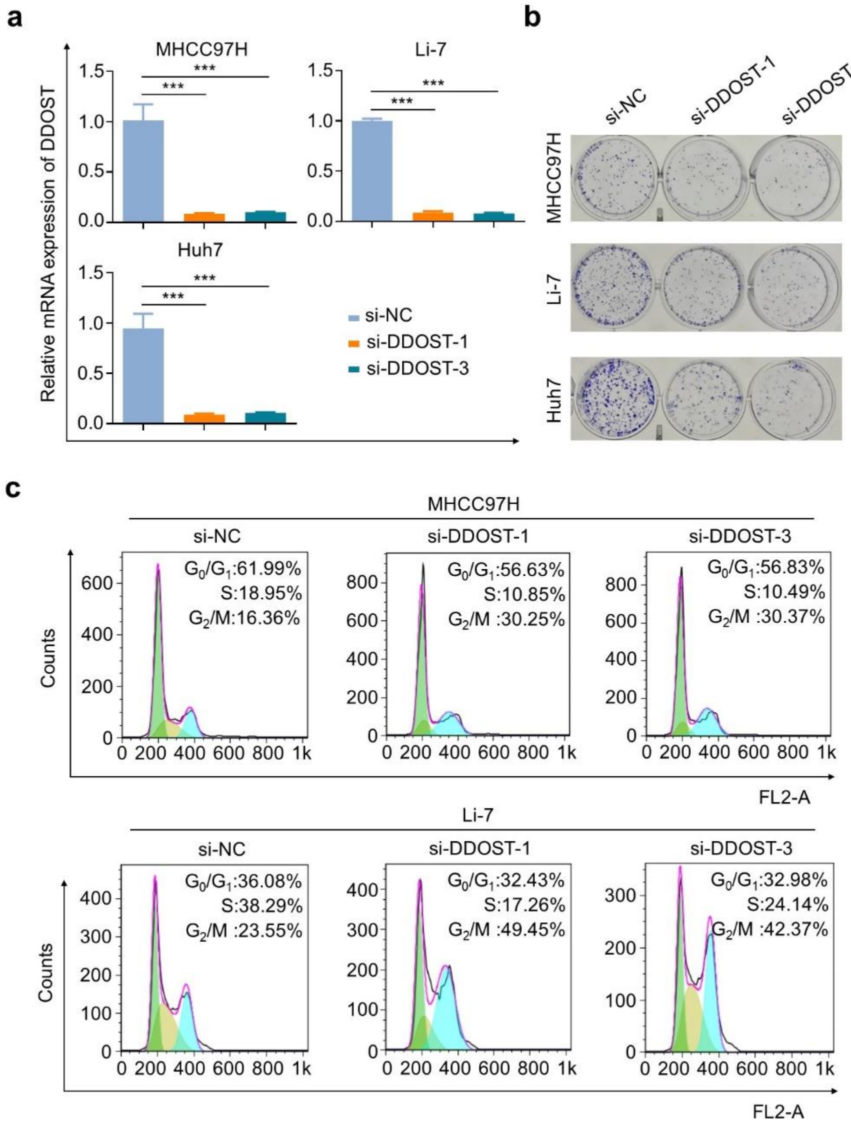

Supplementary Fig. 3 *DDOST* knockdown inhibits HCC cell proliferation. a, qRT-PCR assays were performed to validate the knockdown of *DDOST* by two si-RNAs in MHCC97H and Li-7 cells. *β-Actin* was used as a normalized control. **b**, Representative images of colony formation in the indicated HCC cells. **c**, Flow cytometry analysis demonstrating cell cycle arrest induced by *DDOST* knockdown in MHCC97H and Li-7 cells. Data were expressed as mean ± SD. ***, $P < 0.001$ (unpaired two-tailed Student's t-test for **a**).

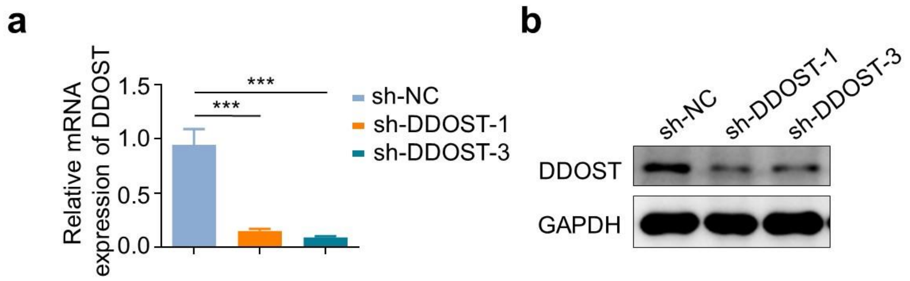

Supplementary Fig. 4 qRT-PCR (**a**) and western blotting (**b**) assays were performed to validate the knockdown of *DDOST* by two sh-RNAs in MHCC97H cells. *β-Actin* was used a normalized control for qRT-PCR and GAPDH was used as a loading control for western blotting analysis. Data were expressed as mean ± SD. ***, P < 0.001 (unpaired two-tailed Student's t-test for **a**).

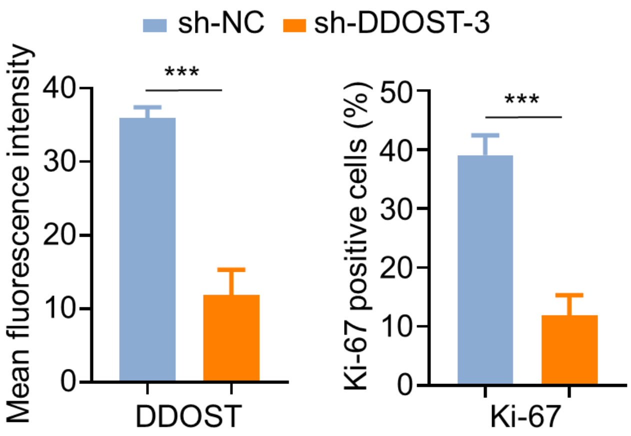

Supplementary Fig. 5 Statistical analysis on IF staining of DDOST and Ki-67 in the indicated tumor tissues (related to Fig. 2h). Data were expressed as mean ± SD. ***, P < 0.001 (unpaired two-tailed Student's t-test).

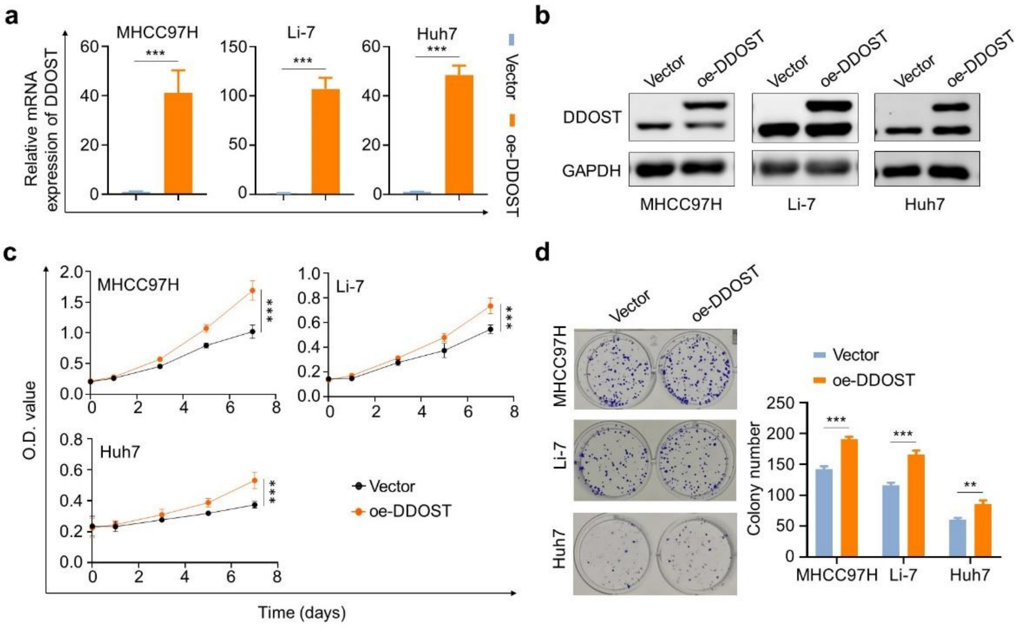

Supplementary Fig. 6 The ectopic expression of *DDOST* promotes HCC cell proliferation. qRT-PCR (**a**) and western blotting (**b**) assays were performed to validate ectopic expression of *DDOST* in MHCC97H, Li-7 and Huh7 cells. *β-Actin* was used a normalized control for qRT-PCR and GAPDH was used as a loading control for western blotting analysis. **c**, The MTT assays showing the effect of *DDOST* overexpression on the proliferation of MHCC97H, Li-7 and Huh7 cells. **d**, Representative images of colony formation in the indicated HCC cells (left panels), and statistical results were presented in the right panel. Data were expressed as mean ± SD. **, $P < 0.01$; ***, $P < 0.001$ (unpaired two tailed Student's t test for **a** and **d,** Two-way ANOVA for **c**).

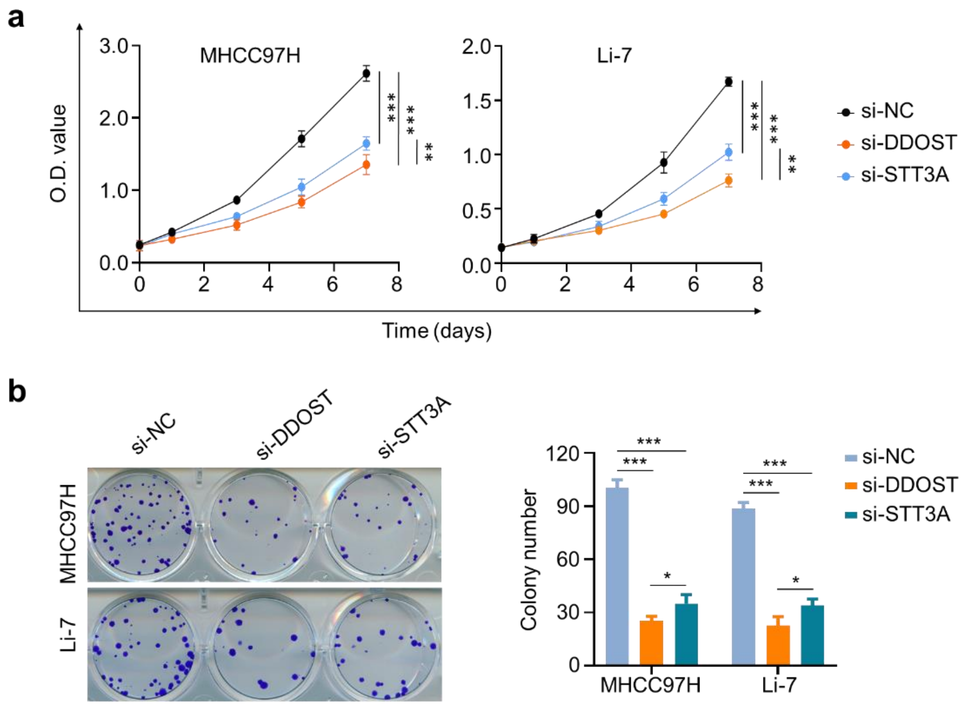

Supplementary Fig. 7 Comparative effects of *DDOST* and *STT3A* knockdown on the growth and colony formation of HCC cells. MHCC97H and Li-7 cells with *DDOST* or *STT3A* knockdown (or their control cells) were used in all experiments. **a**, MTT assays showing cell proliferation over time after *DDOST* or *STT3A* knockdown. **b**, Representative images and quantification of colony formation following *DDOST* or *STT3A* knockdown. Data are presented as mean ± SD from at least three independent experiments. *, $P < 0.05$; **, $P < 0.01$; ***, $P < 0.001$. Two-way ANOVA was used for panels (**a**); unpaired two-tailed Student's t-test was used for panels (**b**).

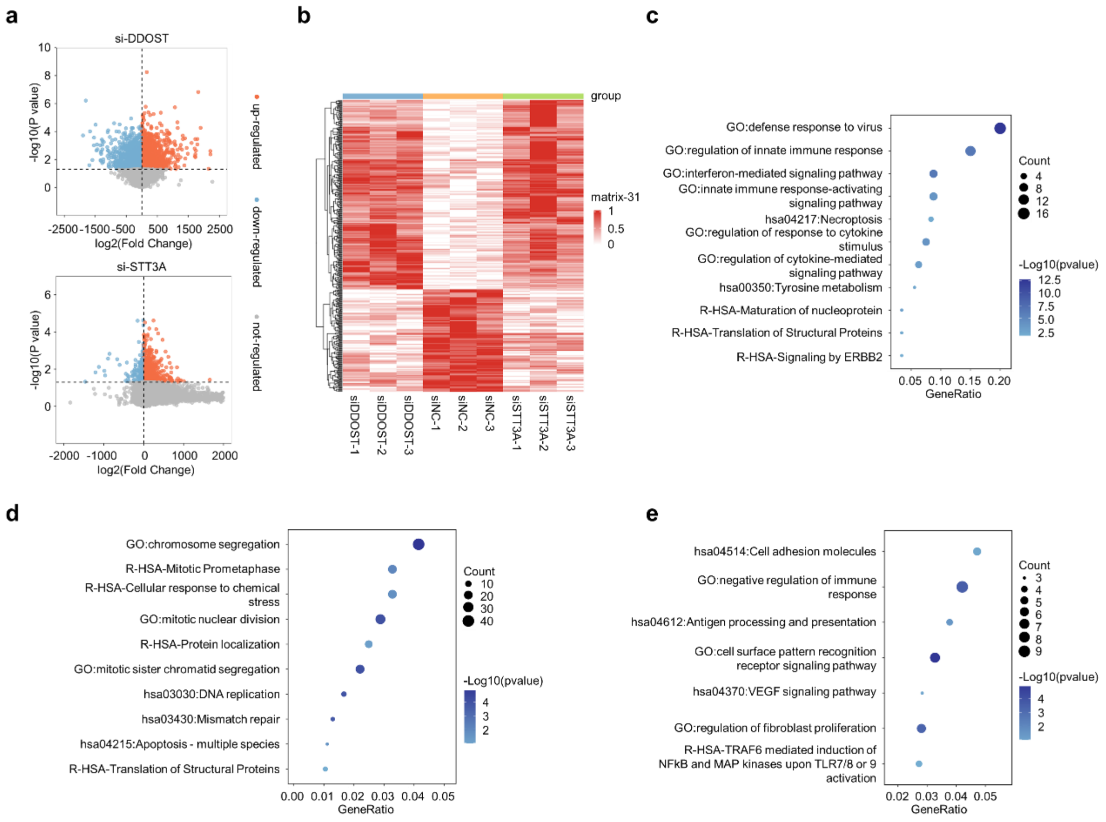

Supplementary Fig. 8 *DDOST* and *STT3A* are involved in the regulation of signaling pathways in MHCC97H cells. a Volcano plots of differentially expressed genes (DEGs) in MHCC97H cells after *DDOST* or *STT3A* knockdown. DEGs were defined as |log2 Fold Change | > 2 and $P < 0.05$. **b** Heatmap of genes that are commonly up-regulated or down-regulated in both *DDOST*- and *STT3A*-knockdown cells (|log2 Fold Change | > 2, $P < 0.05$). Rows indicate genes and columns indicate samples/replicates. **c** Dot plot showing the significantly enriched pathways of down-regulated genes in both *DDOST*-knockdown and *STT3A*-knockdown MHCC97H cells. Log2 |Fold Change | > 2, $P < 0.05$. **d** Dot plot of significantly enriched pathways among genes down-regulated in *DDOST*-knockdown cells (|log2 Fold Change | > 2, $P < 0.05$). **e** Dot plot of significantly enriched pathways among genes down-regulated uniquely in *STT3A*-knockdown cells (|log2 Fold Change | > 2, $P < 0.05$).

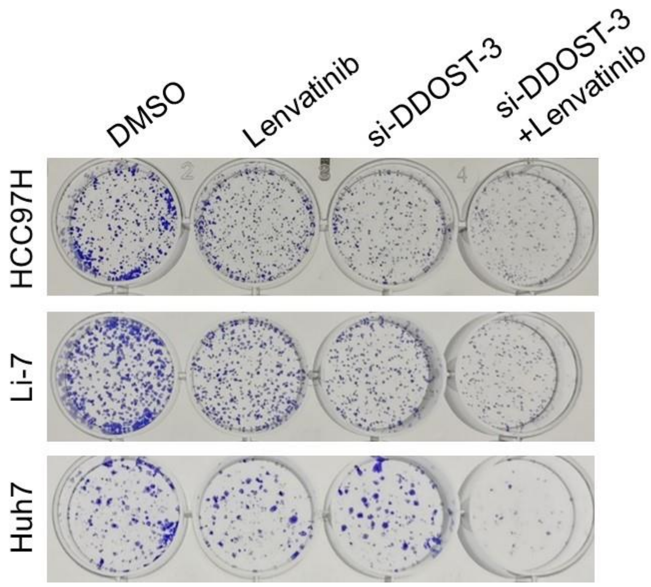

Supplementary Fig. 9 Representative images of colony formation in MHCC97H, Li-7 and Huh7 cells with the indicated treatments (related to Fig. 3c).

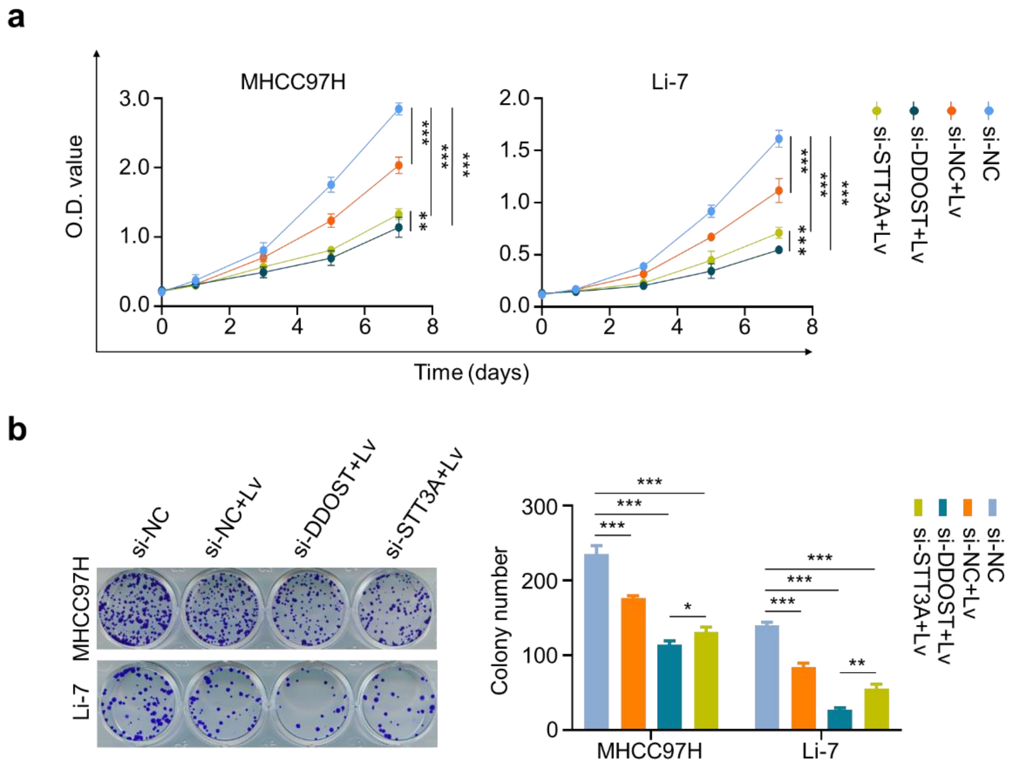

Supplementary Fig. 10 Comparative effects of *DDOST* and *STT3A* knockdown on the lenvatinib sensitivity of HCC cells. MHCC97H and Li-7 cells with *DDOST* or

STT3A knockdown (or their control cells) were used in all experiments. **a**, MTT assays assessing proliferation of *DDOST*- or *STT3A*-knockdown cells and controls after treatment with lenvatinib (as indicated). **b**, Representative colony formation assays and quantification for cells treated as in (**a**). Lv, lenvatinib. Data are presented as mean ± SD from at least three independent experiments. *, $P < 0.05$; **, $P < 0.01$; ***, $P < 0.001$. Two-way ANOVA was used for panels (**a**); unpaired two-tailed Student's t-test was used for panels (**b**).

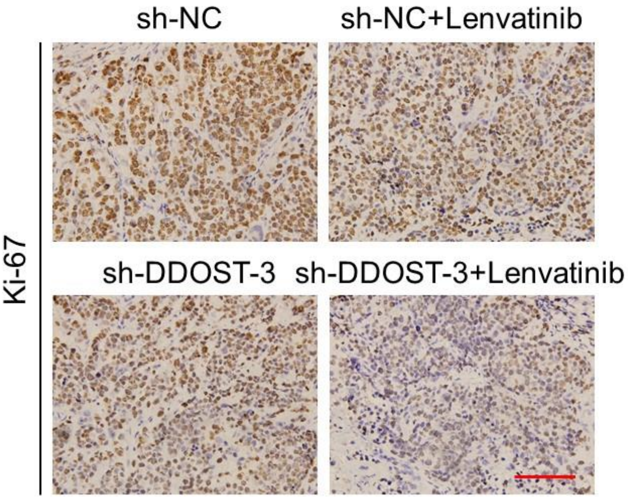

Supplementary Fig. 11 IHC staining of Ki-67 in the indicated tumor tissues (related to Fig. 3g). Scale bar: 100 μm.

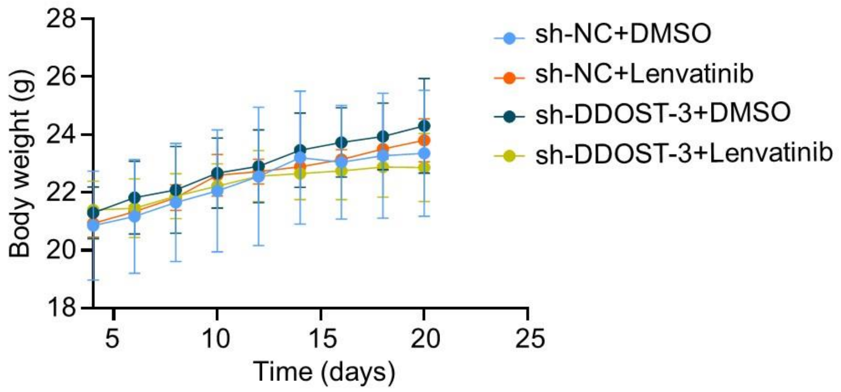

Supplementary Fig. 12 Body weights of mice with the indicated treatments (n = 5 per group). Data were expressed as mean ± SD.

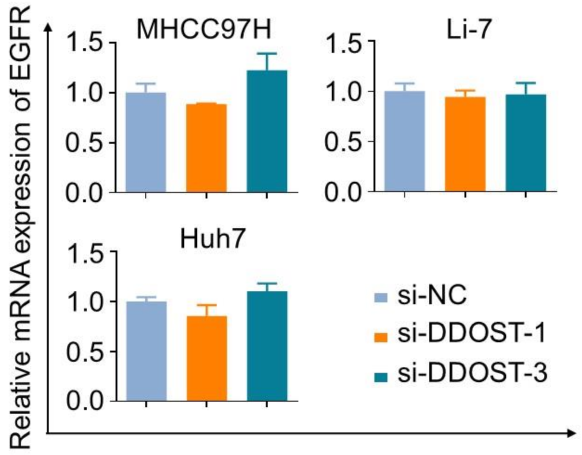

Supplementary Fig. 13 qRT-PCR assays showing the effect of *DDOST* knockdown on mRNA levels of *EGFR* in MHCC97H, Li-7 and Huh7 cells. *β-Actin* was used as a normalized control. Data were expressed as mean ± SD.

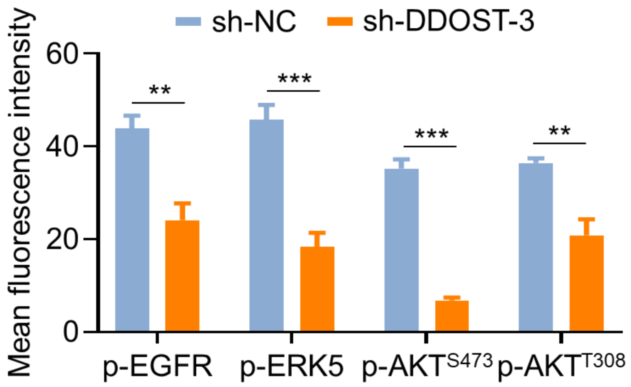

Supplementary Fig. 14 Statistical analysis for IF staining of p-EGFR, p-ERK5 and p-AKT in the indicated tumor tissues (related to Fig. 4l). Data were expressed as mean ± SD. **, $P < 0.01$; ***, $P < 0.001$ (unpaired two-tailed Student's t-test).

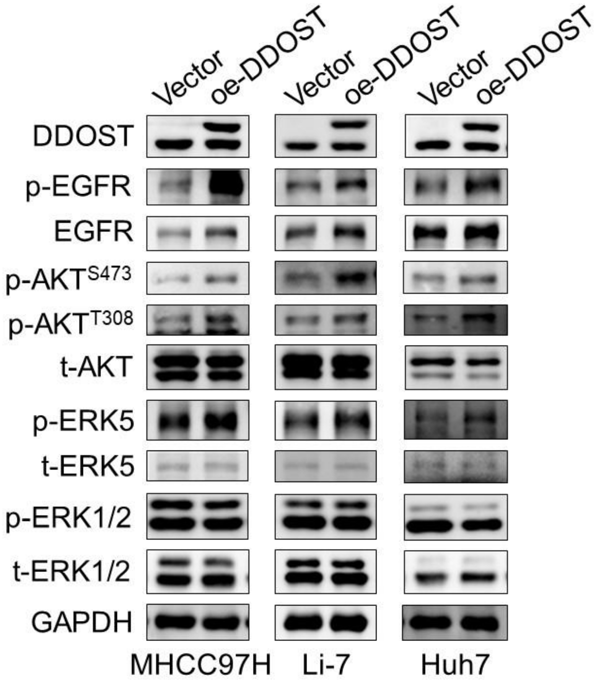

Supplementary Fig. 15 Western blotting analysis showing the effect of DDOST overexpression on the activities of EGFR-mediated signaling pathways in MHCC97H, Li-7 and Huh7 cells. GAPDH was used as a loading control.

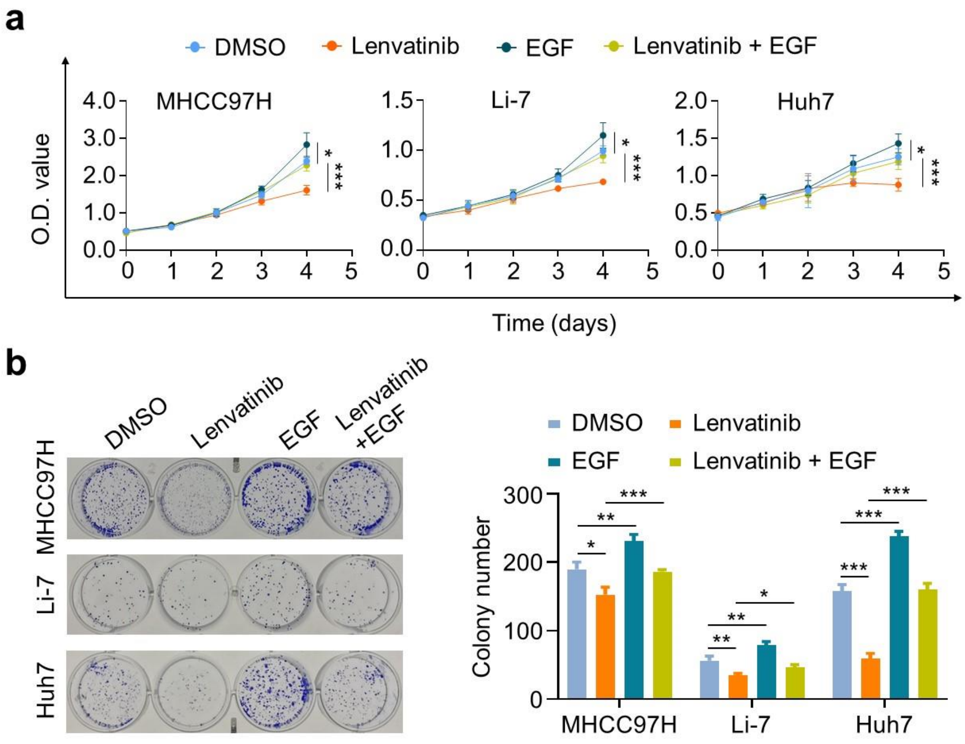

Supplementary Fig. 16 The effects of exogenous EGF on the response of HCC cells to lenvatinib. a, MTT assay showing the effect of EGF and lenvatinib, individually or in combination, on the proliferation of MHCC97H, Li-7 and Huh7 cells. **b**, Representative images of colony formation in MHCC97H, Li-7 and Huh7 cells with the indicated treatments (left panels), and statistical results were presented in the right panel. Data were expressed as mean ± SD. *, $P < 0.05$; **, $P < 0.01$; ***, $P < 0.001$ (Two-way ANOVA for **a**, unpaired two tailed Student's t test for **b**).

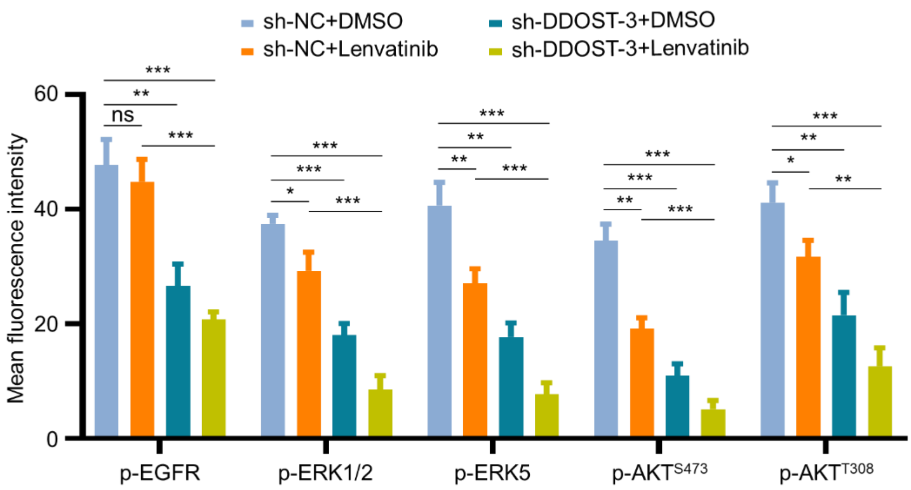

Supplementary Fig. 17 Statistical results on IF staining of p-EGFR, p-ERK1/2, p-ERK5 and p-AKT in the indicated tumor tissues (related to Fig. 4o). Data were expressed as mean ± SD. *, $P < 0.05$; **, $P < 0.01$; ***, $P < 0.001$; ns, no significant (unpaired two-tailed Student's t-test).

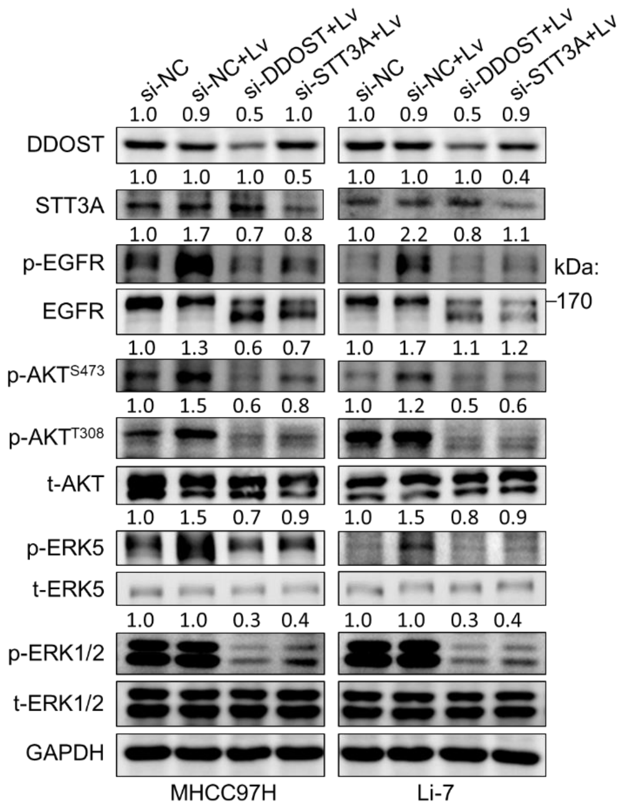

Supplementary Fig. 18 Comparative effects of *DDOST* and *STT3A* knockdown on the activity of EGFR downstream signaling upon lenvatinib treatment in HCC cells. Western blotting analysis of EGFR, p-EGFR, AKT, p-AKT, ERK5, p-ERK5, ERK1/2 and p-ERK1/2 in MHCC97H and Li-7 cells with *DDOST* or *STT3A* knockdown following lenvatinib treatment. Lv, lenvatinib. GAPDH was used as a loading control.

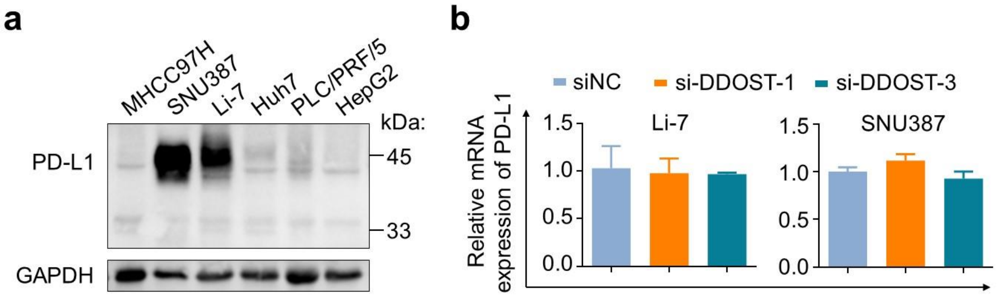

Supplementary Fig. 19 The effect of *DDOST* knockdown on PD-L1 expression in HCC cells. a, Western blotting assay showing PD-L1 expression in a panel of HCC cell lines. GAPDH was used as a loading control. **b**, qRT-PCR assay showing the effect of *DDOST* knockdown on mRNA expression of *PD-L1* in the indicated HCC cells. *β-Actin* was used as a normalized control.

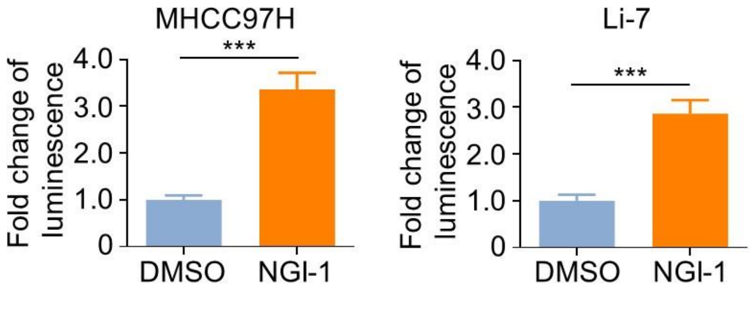

Supplementary Fig. 20 Luminescence assays evaluating the effect of NGI-1 on N-glycosylation of ER-LucT fusion protein in MHCC97H and Li-7 cells. Data were

expressed as mean ± SD. ***, $P < 0.001$ (unpaired two-tailed Student's t-test).

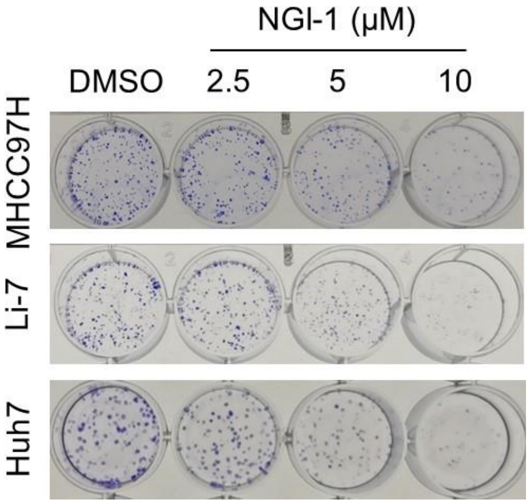

Supplementary Fig. 21 Representative images of colony formation in MHCC97H, Li-7 and Huh7 cells treated with different does of NGI-1 (related to Fig. 6b).

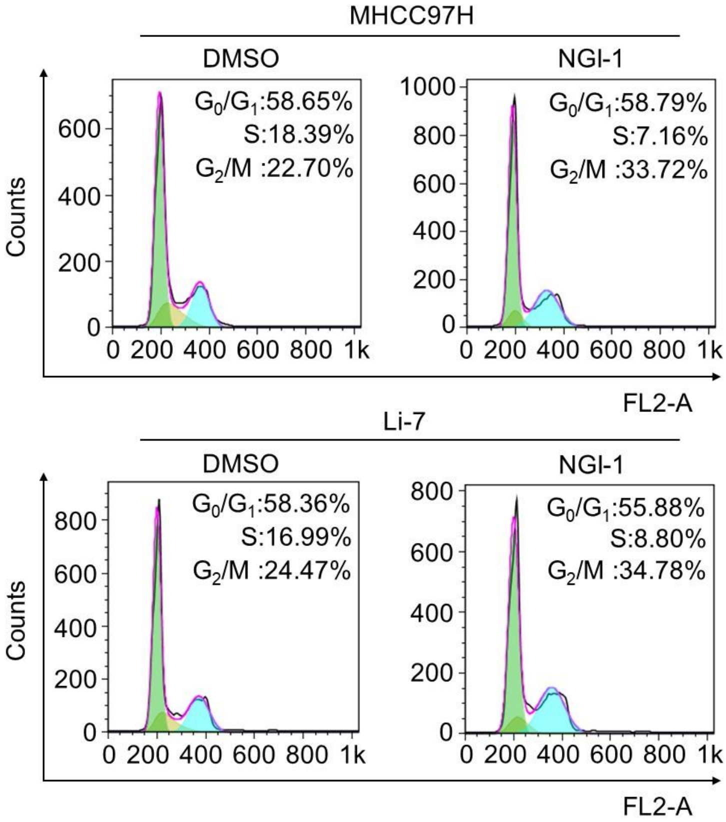

Supplementary Fig. 22 Flow cytometry analysis demonstrating cell cycle arrest

induced by NGI-1 in MHCC97H and Li-7 cells (related to Fig. 6c).

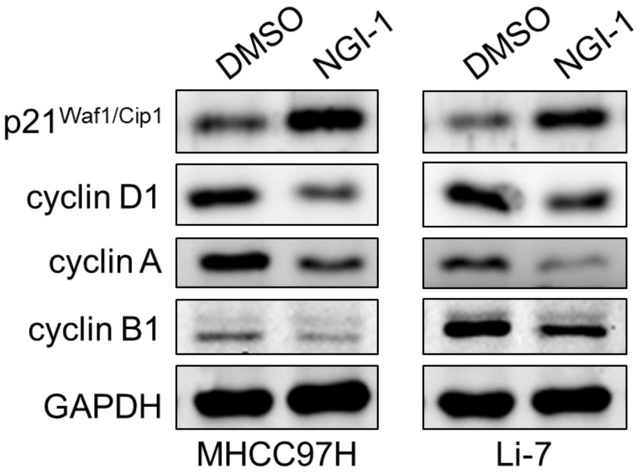

Supplementary Fig. 23 Western blotting analysis evaluating the expression of key proteins associated with cell cycle transition in MHCC97H and Li-7 cells treated with or without NGI-1. GAPDH was used as a loading control.

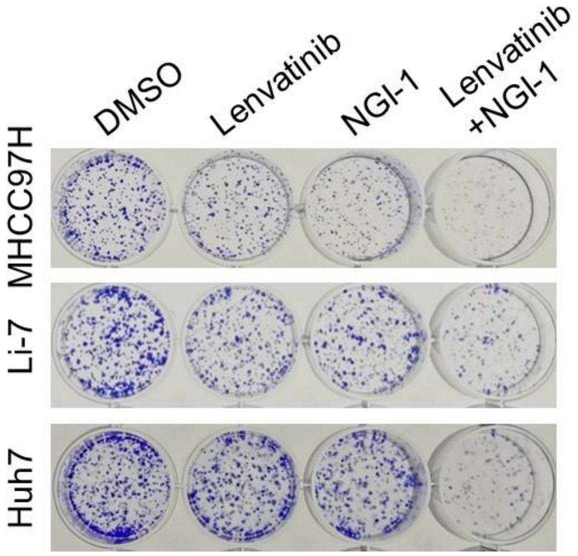

Supplementary Fig. 24 Representative images of colony formation in MHCC97H, Li-7 and Huh7 cells treated with lenvatinib and NGI-1, individually or in combination (related to Fig. 6f).

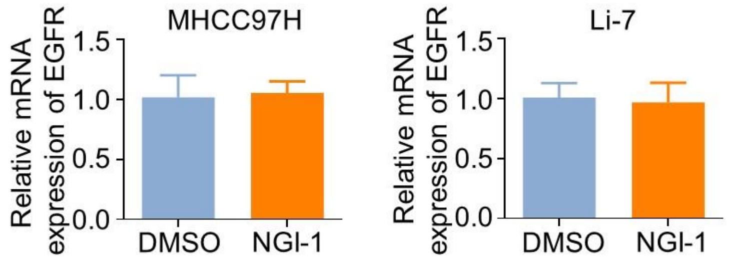

Supplementary Fig. 25 qRT-PCR assays showing the effect of NGI-1 on mRNA expression of *EGFR* in MHCC97H and Li-7 cells. *β-Actin* was used as a normalized control. Data were expressed as mean ± SD.

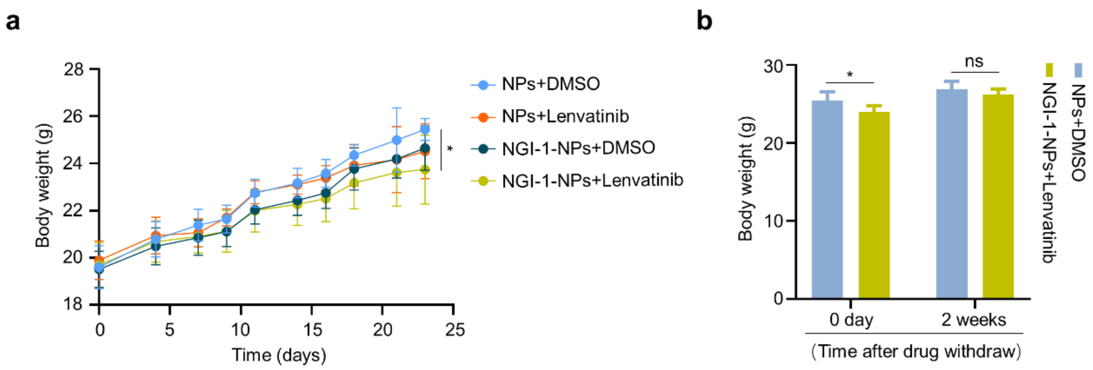

Supplementary Fig. 26 Body weights of mice with the indicated treatments. a Body weights of mice with the indicated treatments (n = 5 per group) during the whole experiment. **b** Body weight changes of mice after the indicated treatments (n = 5 per group). Data were expressed as mean ± SD. *, $P < 0.05$; ns, not significant (Two-way ANOVA for **a**, unpaired two-tailed Student's t-test for **b**).

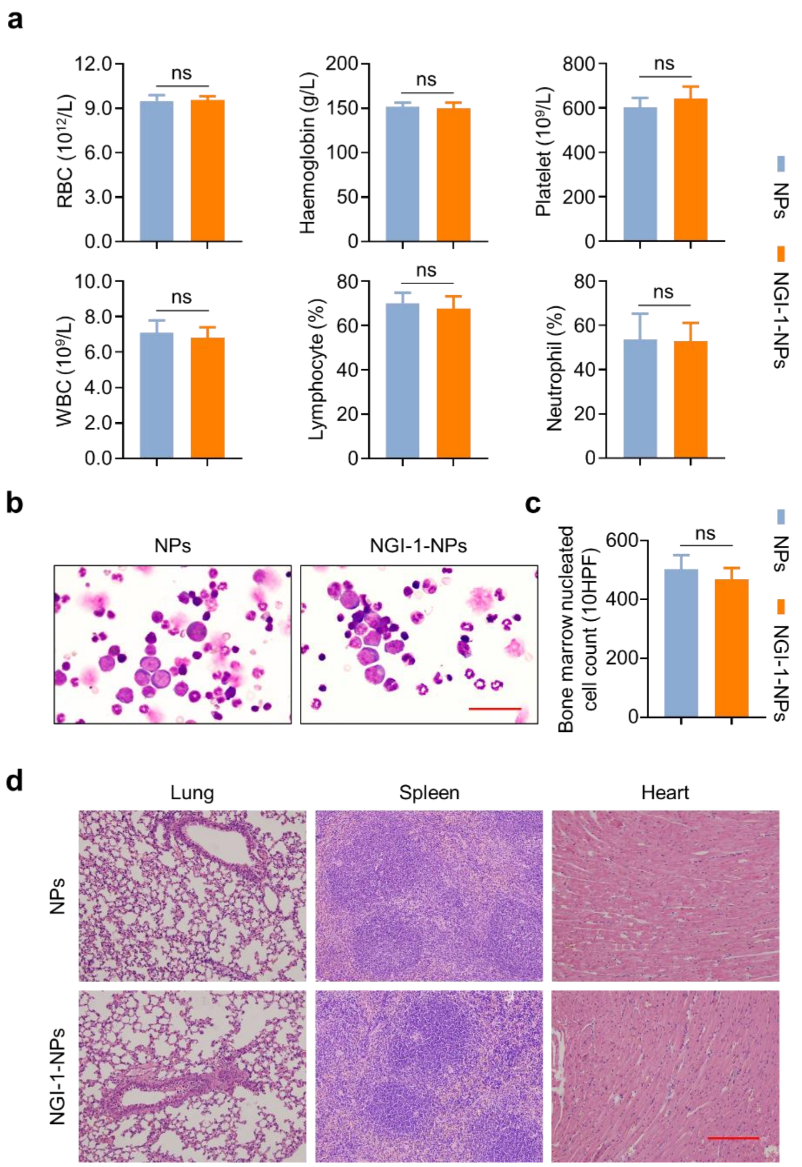

Supplementary Fig. 27 Toxicity assessment of NGI-1-loaded nanoparticles (NGI-1-NPs) in BALB/c mice. a Comparison of peripheral blood cell counts between mice treated with empty NPs and NGI-1-NPs (including WBC, RBC, PLT, etc.). **b** Representative Wright–Giemsa staining of bone marrow smears from each treatment group (1000×). Scale bar: 100 μm. **c** Comparison of bone marrow nucleated cell counts among the indicated treatment groups. **d** Representative H&E staining of lung, spleen and heart from each group (200×). Scale bar: 100 μm. Data were shown as mean ± SD.

*, $P < 0.05$; ns, not significant. Statistical comparisons in panels **a** and **c** were performed using an unpaired two-tailed Student's t test.

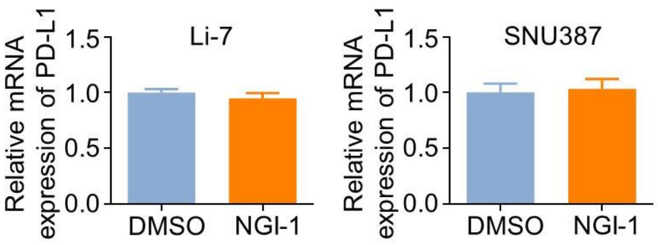

Supplementary Fig. 28 qRT-PCR assays showing the effect of NGI-1 on mRNA expression of *PD-L1* in Li-7 and SNU387 cells. *β-Actin* was used as a normalized control. Data were expressed as mean ± SD.

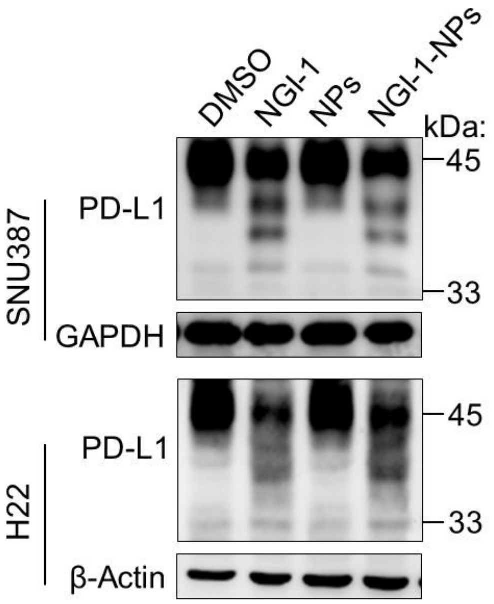

Supplementary Fig. 29 Western blotting analysis showing the effect of NGI-1-NPs on N-glycosylation of PD-L1 in SNU387 and H22 cells. GAPDH and β-Actin were used as the loading controls.

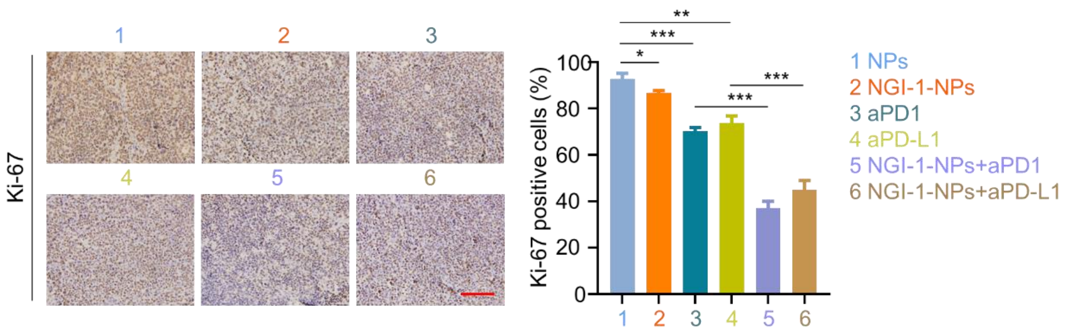

Supplementary Fig. 30 IHC analysis of Ki-67 in the indicated tumor tissues. Left panels show the representative images, and statistical results were presented on the right panel. Scale bar: 100 µm. Data were expressed as mean ± SD. *, $P < 0.05$; **, $P < 0.01$ (unpaired two-tailed Student's t test).

Supplementary Tables

Supplementary **Table 1.** The sequences of primers used in this study

Genes	Forward primer (5'-3')	Reverse primer (5'-3')
DDOST	TTCAAGACCGCTGATGACCC	TCGTCAAACTCAATCCCGCA
β-Actin	GCACAGAGCCTCGCCTT	GTTGTCGACGACGAGCG
EGFR	AACACCCTGGTCTGGAAGTACG	TCGTTGGACAGCCTTCAAGACC
CD274	TGCCGACTACAAGCGAATTACTG	CTGCTTGTCCAGATGACTTCGG

Supplementary **Table 2.** The antibodies used in this study

Antibodies	Catalog#	Source
anti-DDOST	sc-74408	Santa Cruz
anti-GAPDH	TDY052F	TDYbio
anti-p21	#2947S	Cell Signaling Technology
anti-cyclin A	sc-271682	Santa Cruz
anti-cyclin B1	sc-245	Santa Cruz
anti-cyclin D1	ab134175	Abcam
anti- Ki-67	ab15580	Abcam
anti-EGFR	#4267S	Cell Signaling Technology
anti- p-EGFR (Tyr1068)	#4777S	Cell Signaling Technology
anti-AKT	#4691S	Cell Signaling Technology
anti- p-AKT (Ser473)	#4060L	Cell Signaling Technology
anti- p-AKT (Ser473)	BS9913M	Bioworld
anti- p-AKT (Thr308)	#13038S	Cell Signaling Technology
anti- p-AKT (Thr308)	AP0056	Bioworld
anti-ERK5	sc-398015	Santa Cruz
anti-p-ERK5	sc-135760	Santa Cruz
anti-ERK1/2	#4695S	Cell Signaling Technology
anti-p-ERK1/2	#4370S	Cell Signaling Technology

anti-PD-L1(human)	GTX104763	GeneTex
anti-PD-L1(mouse)	ab213480	Abcam
anti-β-Actin	AP0060	Bioworld
Alexa Fluor 488 Goat anti-rabbit IgG	A32731	Thermo Fisher Scientific
Alexa Fluor 647 Goat anti-rabbit IgG	A21244	Thermo Fisher Scientific
Alexa Fluor 647 Goat anti-mouse IgG	A21235	Thermo Fisher Scientific

Supplementary **Table 3**. The sequences of si-RNAs used in this study

**si-RNAs**	**Sense (5'-3')**	**Antisense (5'-3')**
si-DDOST-1	GCUGGAGUUUGUCCGCATT	AAUGCGGACAAACUCCAGCTT
si-DDOST-2	CCCAAUGCCUACACUGUCATT	UGACAGUGUAGGCAUUGGGTT
si-DDOST-3	GCUUUGAGCUCACAUUCAATT	UUGAAUGUGAGCUCAAAGCTT
si-STT3A	CUGUAGUUCCUGGAUAUAUTT	AUAUAUCCAGGAACUACAGTT
si-NC	UUCUCCGAACGUGUCACGUTT	AAACGTGACACGTTCGGAGAATT

Supplementary **Table 4**. The sequences of sh-RNAs used in this study

**sh-RNAs**	**Top Strand**	**Bottom Strand**
sh-DDOST-1	CCGGCCCTTTGATGGCGATGACATT CTCGAGAATGTCATCGCCATCAAAG GGTTTTTG	AATTCAAAAACCCTTTGATGGCGATG ACTTCTCGAGAATGTCATCGCCATCAA AGGG
sh-DDOST-2	CCGGCTCACATTCAAGACCGCTGAT CTCGAGATCAGCGGTCTTGAATGTG AGTTTTTG	AATTCAAAAACTCACATTCAAGACCC TATCTCGAGATCAGCGGTCTTGAATGT GAG
sh-DDOST-3	CCGGGACAAGCCTATCACCCAGTAT CTCGAGATACTGGGTGATAGGCTTG TCTTTTTG	AATTCAAAAAGACAAGCCTATCACCA GATCTCGAGATACTGGGTGATAGGCTT GTC
sh-NC	CCGGTTCTCCGAACGTGTCACGTCT	AATTCAAAAATTCTCCGAACGTGTCA

	CGAGACGTGACACGTTCGGAGAATT TTTG	CGTCTCGAGACGTGACACGTTCGGAG AA
